# How decarbonization and the circular economy interact: Benefits and trade-offs in the case of the buildings, transport, and electricity sectors in Austria

**DOI:** 10.1111/jiec.13619

**Published:** 2025-01-31

**Authors:** W. Haas, A. Baumgart, N. Eisenmenger, D. Virág, G. Kalt, M. Sommer, K. Kratena, I. Meyer

**Affiliations:** 1 Institute of Social Ecology, University of Natural Resources and Life Sciences (BOKU), Vienna, Austria; 2 Austrian Institute of Economic Research (WIFO), Vienna, Austria; 3 Centre of Economic Scenario Analysis and Research (CESAR), Vienna, Austria

**Keywords:** circular economy, decarbonization, land take, narrowing loops, policy targets, socio-ecological transformation

## Abstract

The widely heralded decarbonization of economies is a significant intervention in countries’ societal metabolism, which eliminates the use of fossil fuels but also requires renewing societal stocks such as buildings, vehicles, and power plants, which in turn requires materials and energy. The circular economy (CE) shifts a country’s metabolism toward less material demand, waste, and emissions, moving away from a linear resource flow pattern to one that narrows and slows flows and closes loops, in order to support climate protection. This article uses the example of Austria to examine how decarbonization and CE interact in the buildings, transport, and electricity sectors. We use scenarios to analyze the contribution of decarbonization and CE strategies to achieve targets set by Austrian policy: (1) carbon neutrality by 2040, (2) ambitious reductions in material consumption, and (3) limiting annual land take. A scenario focusing on “decarbonization” alone reduces processed materials by 7% compared to the reference scenario, but is associated with high risks: it requires large supplies of green electricity, technology-critical elements, and smooth permitting procedures. A “weak CE” scenario shows little mitigating effects on these risks. CE and land take targets are missed in the two scenarios. Avoiding further expansion of buildings and roads on unbuilt land as part of a “strong CE” scenario is identified as key to narrow the processed materials of respective sectors from 102 to 26 Mt/a consistent with all three policy targets. It reduces inter alia demand for green electricity facilitating decarbonization and additionally generating co-benefits for health.

**Supplementary Information:**

The online version of this article (doi:10.1111/jiec.13619) contains supplementary material, which is available to authorized users.

## INTRODUCTION

### Decarbonization in interaction with the circular economy

With the Paris Agreement on climate change, 195 countries agreed to limit global warming (United Nations, 2015). Meanwhile, many countries have committed themselves to achieving carbon neutrality by 2040 (Austria), 2050 (EU and United States), or 2060 (China) (Zhao et al., 2022). Achieving this goal is challenging, as sufficient green power plants are needed, the heating and cooling of buildings must be decarbonized and the transport, agricultural, and industrial sectors must gradually phase out fossil fuels.

Such a far-reaching transformation requires a major rebuilding of the material stocks of economic sectors, which in turn will require materials and energy in the coming decades. The challenges to such a swift transformation are manifold: There may be a shortage of skilled labor (Briggs et al., 2022). A smooth and timely process to achieve the agreed targets will require rapid approval procedures for new energy infrastructure (Hübner et al., 2023). Further, large amounts of bulk materials are demanded (Kalt et al., 2022; Wang et al., 2023), and there may be supply bottlenecks for scarce materials (C. Zhang et al., 2023).

The Global Resource Outlook (UNEP IRP, 2024) emphasizes that the increasing use of resources is the main cause of the triple planetary crisis, namely climate change, environmental pollution, and biodiversity loss. Thus, there are clear limits to the current high levels of resource use in industrialized countries which prompted some governments to set goals for a reduction of the per capita resource use.

Against this backdrop, the concept of a circular economy (CE) has gained prominence, as it offers multiple promises, amongst them to reduce material inputs and wastes while at the same time supporting climate change mitigation (Cantzler et al., 2020). However, these potentials remain understudied (Gallego-Schmid et al., 2020). In a systematic review of the mitigation potential of CE strategies, only 10% of studies on CE tackled its climate mitigation potential (Cantzler et al., 2020). However, some scholars have suggested that cyclical systems can still consume resources, generate waste, and emit carbon due to the energy required to operate (Corvellec et al., 2022), and that recycling is not enough to make the world a greener place (Guo et al., 2023).

Since decarbonization processes are on the way, a broader perspective with a critical view of the potential of CE strategies becomes crucial (Ghorbani et al., 2024). It is essential to identify the specific CE strategies, from narrowing, slowing, to closing loops, that have the potential to successfully facilitate decarbonization while reducing demand for primary resources, polluting outflows to nature, and adverse impacts on biodiversity.

As such an investigation can reveal more specific insights in the concrete context of a case, we investigate the Austrian biophysical economy in its complex interwovenness of materials and energy, as well as stocks and flows. In addition, Austria serves as a good example due to its established, concrete CE and decarbonization targets.

### Austria’s biophysical economy and policy targets

**FIGURE 1:**
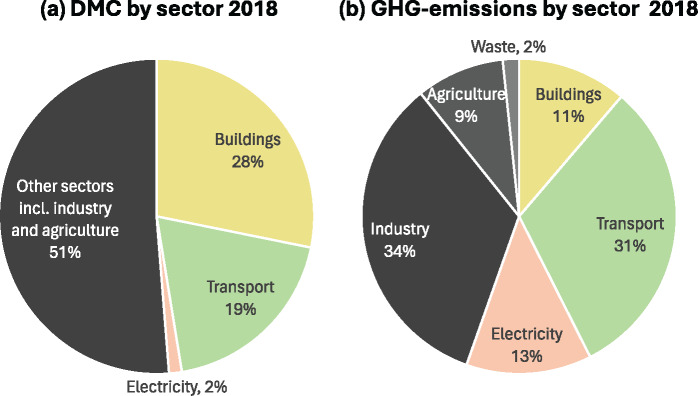
Sector shares in (a) domestic material consumption (DMC) (results from the circular economy Austria model) and (b) Austrian territorial greenhouse gas emissions (based on UBA, 2022) in Austria in 2018 (underlying data are available in SI-data).

Austria’s domestic material consumption (DMC), which is domestic extraction (DE) plus imports minus exports (Krausmann et al., 2015), was 161 Mt/a in 2018. The buildings, transport, and electricity sectors collectively accounted for 74 Mt/a representing 49% of DMC in 2018, of which 28% (47 Mt/a) are used for the buildings sector, 19% (36 Mt/a) for the transport sector, and 2% (5 Mt/a) for the electricity sector, leaving more than half of DMC for other sectors including industry and agriculture (Figure 1a). In terms of greenhouse gas (GHG) emissions, these three sectors collectively contributed to slightly more than half of total GHG emissions in Austria in 2018 (Figure 1b).

The current Austrian government program entails the vision of making Austria carbon neutral by 2040 (BKA, 2020). The Austrian national action plan for climate and energy (BMNT & BMVIT, 2018) defines further targets for decarbonization until 2030. To achieve that, reductions of 7 MtCO_2e_ in the transport sector and 3 MtCO_2e_ in the buildings sector (compared to 2016) are planned. A special feature of the Austrian electricity sector is the high share of hydropower (run-of-river and storage power plants), which accounted for 58% in 2018. By 2030, electricity shall be provided entirely from renewable energy, and 46%–50% of the gross final energy use shall be generated from renewable resources. In the transport sector, a minimum of 14% of energy shall be generated from renewable resources (BMNT, 2019).

A CE strategy approved by the government at the end of 2022 sets the goal of reducing the DMC from 18 t/cap/a in 2018 to 14 t/cap/a by 2030 and material footprint to 7 t/cap/a by 2050. An input circularity rate (representing the share of recycled materials in DMC including recycling) of 18% is aimed at in 2030 (BMK, 2022). The Austrian government program states that a target path to reduce land take to net 2.5 ha/day by 2030 is to be implemented (BKA, 2020).

The goals set by the Austrian government form the benchmark for our analysis of decarbonization and CE scenarios. Herein, we focus on the buildings, transport, and electricity sector as they are supposed to have high carbon saving potentials (Cantzler et al., 2020).

Our research question is focused on how decarbonization and CE strategies interact, that is, to what degree certain combinations of decarbonization and specific narrowing, slowing, and loop-closing strategies reduce material and energy, respectively, C emissions over time including what benefits and trade-offs are associated with these combinations.

## METHODS

Our analysis builds on a CE stock-flow model, which traces all material and energy flows from inputs to outputs through the socioeconomic system in a mass-balanced approach. The model was first developed for a global CE assessment (Haas et al., 2015, further developed in Haas et al., 2020), then applied to Austria for 2014 including a sensitivity analysis for testing the robustness of data (De Wit et al., 2019; Jacobi et al., 2018), to the EU (Mayer et al., 2019), and most recently to South Africa (Haas et al., 2023). In the research presented herein, the Austrian model (circular economy Austria; CeAT) was updated to 2018 and then used to first provide reference scenarios of material and energy flows until 2040, and second provide three scenarios implementing further decarbonization and CE measures. As we started our analysis during the first year of the COVID-19 pandemic, there was high uncertainty about the future economic development of the country, and a plain continuation of past GDP trends seemed unrealistic. We therefore developed two economic projections. In the reference scenario “smooth recovery” (R1), the economy recovers after a short pandemic-caused dip of gross domestic product (GDP) and grows at an average annual rate of 1.3%. In the reference scenario “slow recovery, zero growth” (R2), the economy is only recovering slowly after the pandemic and GDP growth stagnates (see SI section 1.1).

For the reference scenarios we calculated historic material intensity trends of GDP (t/€) of the last 15 years (2004–2018) for DE, imports, and exports of the main material categories. This trend analysis results in slightly improving material intensities (see SI Figure SI-2). By multiplying these material intensities with the projected GDP, we calculated material and energy flows of DE, imports, and exports, and thereof DMC for every year until 2040 for both reference scenarios (Figure SI-3, equations listed in Table SI-1).

**FIGURE 2:**
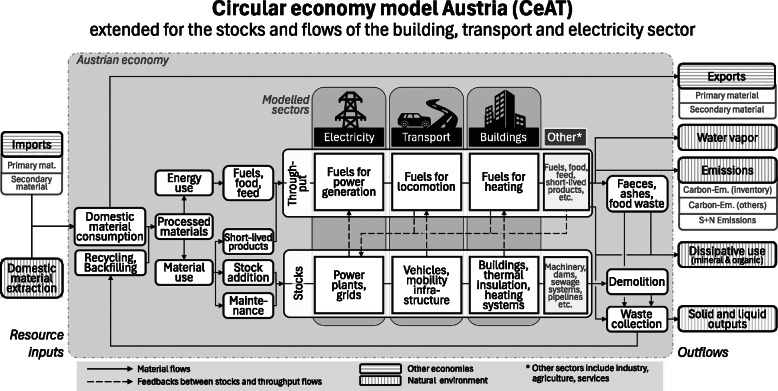
Basic structure of the model circular economy Austria.

As societal stocks are the primary drivers of material consumption (Krausmann et al., 2017, 2020), the modeling of decarbonization and CE measures requires to explicitly consider and integrate material stocks. Therefore, we extended the CeAT model by introducing material stocks for the three sectors of observation: buildings, transport, and electricity. All other sectors are included in an unspecific category “other” (Figure 2), where no change in relation to the reference scenarios is realized. The development in the three focal sectors was designed in separate modules by implementing alterations of stocks (including buildings, insulation, heating/cooling systems, roads, rails, vehicles, charging stations, power plants, grids, and so on) as deviation from the reference scenarios for scenario-specific decarbonization and CE measures. These stock changes then directly alter stock addition and demolition in the CeAT model. In addition, stock changes have feedback on throughput flows (see Figure 2) within and between the modules. As an example, a certain mix of conventional and electric vehicles demands throughputs of fuels and electricity for the respective mileage determined within the scenarios. The electricity demand in turn has feedback on power generation capacities which then changes inflows of fuel demand.

Input data to the CeAT model (i.e., imports, DMC) were derived from Eurostat and a previous study (Eurostat, 2020a, 2020b, 2021b; Krausmann et al., 2018). We estimated material stocks for the three sectors based on different sources (see SI) and related fuel, respectively, electricity demand for the operation of the relevant stocks through official factors as used in GHG inventories (SI section 1). In total, we distinguished between 28 materials for 76 stock types, which we split according to various quality characteristics (e.g., type single family house is split into age cohorts) (SI section 1.1.4).

Three prospective decarbonization and CE scenarios were developed with increasing intensity of strategies for the three sectors. The scenarios have the following guiding storyline.

- **R: Reference**: The government is continuing the pathway with only moderate changes as they were already underway in previous years without pursuing carbon neutrality or CE targets (with existing measures).
- **A: Decarbonization**: The government is implementing the necessary strategies to achieve its goal of carbon neutrality by 2040 as set out in the coalition agreement (BKA, 2020). No additional CE measures are introduced.
- **B: Decarbonization and weak CE strategies**: In addition to scenario A, all concrete measures as stated in official strategy documents, scenario, or research studies that can be considered as narrowing, slowing, and closing loops.
- **C: Decarbonization and strong CE strategies**: In addition to measures in scenario A and going beyond CE measures in scenario B, a range of far-reaching CE strategies, including sufficiency strategies, which aim first at narrowing, then at slowing, and finally at closing of loops.

The packages of measures designed for scenarios B and C are based on first, planned interventions, which are sketched out in relevant policy documents (Baumann & Kalt, 2015; BMK, 2021, 2022; BMNT, 2019; UBA, 2023), and second, scientific findings on the most effective CE and decarbonization strategies. For example, a shift toward renewable energy, eco-design, and reductions of societal material stocks have been found most relevant to advance circularity (Haas et al., 2015; Miatto et al., 2024). The specific measures assumed in each scenario are summarized in Table Table 1.

**TABLE 1:** Overview of circular economy measures in the three scenarios for the three sectors.

Sector	Measure	A Decarbonization	B Decarbonization and “weak CE”	C Decarbonization and “strong CE”	Narrowing	Slowing	Loop closing
Buildings	Decarbonization of heating/cooling systems	100% phase out of fossil fuels in heating/cooling by 2040			**•**		
	Improved thermal renovation rate	Starting with 1.3% of the building stock in 2018; increasing to 2.1% in 2030			**•**		
	Increased insulation in new buildings	∼75% increase in insulation material thickness in new construction			**•**		
	Reduction of floor space in new buildings^a^	No restriction	25% reduction in per capita floor area^b^	No construction on unbuilt land after 2030 limits floor space in new buildings	**•**		
	Lifetime extension of buildings	No lifetime extension measures		Demolition reduced by 25%^b^		**•**	
	Increased timber construction share	Current shares of timber in construction continued		Share of timber construction in total construction steadily increased to 50% by 2040	**•**		
Transport	Fleet electrification	100% electrification of the vehicle fleet by 2040			**•**		
	Traffic volume reduction by 2040^b^	No reduction	−15% pkm −25% tkm	−30% pkm, −50% tkm	**•**		
	Modal split shift by 2040^b^	−10% PPV pkm^c^ −10% tkm road	−20% PPV pkm^c^ −20% tkm road	−50% PPV pkm^c^ −40% tkm road	**•**		
	Car fleet size reduction	No reduction	Reduced in proportion to pkm/tkm		**•**		
	Car sharing	Only up to about 0.01% of cars are shared		Two users share one car by 2040	**•**		
	Limiting road network development	No limitation of road construction		Expansion halt by 2030	**•**		
Electricity	Fossil fuel phase out ▪ Hydro capacity constant in GW in all scenarios ▪ Biomass to power declines in B and C from 2018 ▪ Installed capacities for all scenarios account for change in electricity demand for buildings and transport	Wind: 23% PV: 25% Hydro ROR: 34% Hydro-storage: 11% Biomass: 5% Waste: 1%	Wind: 21% PV: 24% Hydro ROR: 37% Hydro-storage: 12%, Biomass: 5%, Waste: 1%	Wind: 18%, PV: 20%, Hydro ROR: 42% Hydro-storage: 14% Biomass: 4% Waste: 1%	**•**		
Economy wide	Increased recycling rates	Iron/steel: 79% Plastics: 30%, Timber: 72% Constr. minerals: 92%	Iron/steel: 79% Plastics: 36% Timber: 72% Constr. minerals: 92%	Iron/steel: 86% Plastics: 57% Timber: 81% Constr. minerals: 95%			**•**

Please refer to Table SI-4 for data sources.

Abbreviations: GW, gigawatt; pkm, person kilometer; PPV, private passenger vehicles; PV, photovoltaic; ROR, run of river; tkm, ton kilometer.

^a^Floor space refers to the total floor area. A reduction in overall floor space implies a reduction in heated floor space (proportionally scaled).

^b^Compared to reference scenario.

^c^PPV pkm allocated to public transport and active mobility based on reference scenario shares.

The majority of materials covered in this study are bulk materials used en masse. To also gain insights into the opposite end of the spectrum, we quantified in a back-of-the-envelope calculation changes in the annual material demand of technology-critical materials in vehicles, photovoltaic (PV), and wind turbines based on factors compiled by Baumgart et al. (2024) (SI section 1.4.4).

The translation of the large pool of decarbonization and CE measures to a manageable package that can be implemented in the CeAT model in a mass-balanced way is an ambitious task with some limitations: Scenarios do not consider increase in future climate change impacts and the material required for damage repair, only material intensity trends based on past developments for main material categories are assumed, and a split of main material categories into detailed material flows follows fixed ratios from the past. Further, feasibility of increased public transport without extending transport networks and high usage of heat pumps is assumed without in-depth investigation. Additionally, refurbishing flows for buildings with lifetime extensions are not explicitly considered, as we assume this to be accounted for in thermal renovation material flows. However, we are of the opinion that these limitations do not jeopardize the robustness of the results as they apply equally to all scenarios and thus do not significantly distort the relation between the scenarios or they refer to relatively small flows, leading to uncertain but nevertheless minimal impacts on the economy-wide findings (SI section 1.6).

## RESULTS

### The scenarios’ material demand in 2040

**FIGURE 3:**
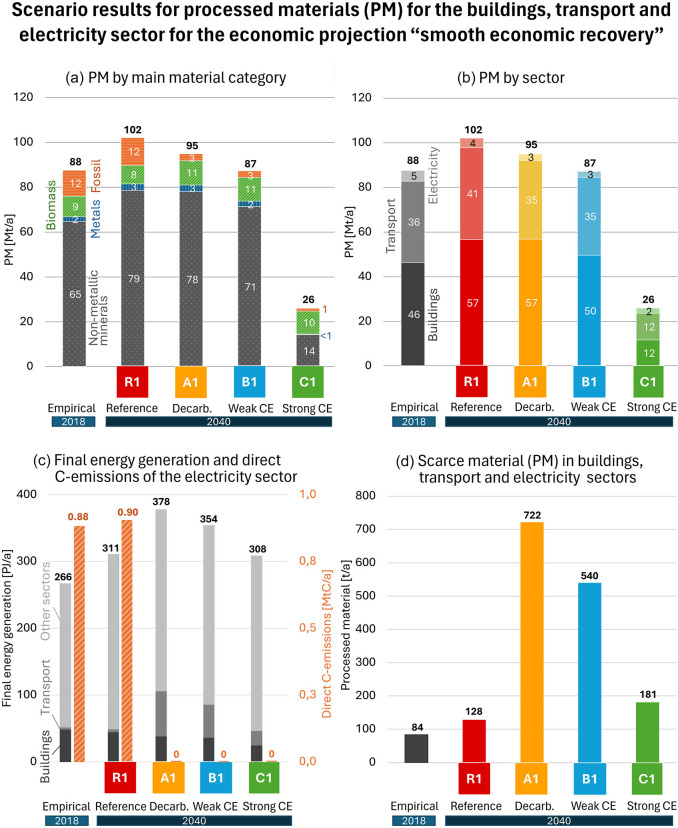
All graphs show results for 2018 and four scenarios for buildings, transport, and electricity sector for the economic projection smooth economic recovery for 2040: (a) shows the processed material (PM) break down into the four material categories; (b) displays the sector split for buildings, transport, and electricity; (c) presents the final energy generation and direct C emissions of the electricity sector considering change in electricity demand in buildings and transport sector; and (d) indicates the demand for scarce materials (underlying data are available in SI-data).

For Austria in the year 2040, we have calculated the processed materials (PM = DMC + Recycling) for the three sectors: buildings, transport, and electricity production for all scenarios. First, we present the results for the economic projection under a smooth economic recovery (index 1) (Figure 3). The reference scenario R1 shows an increasing PM from 88 Mt/a in 2018 to 102 Mt/a (+16%) in 2040.

When we introduce a full decarbonization for the three sectors (A1), PM is reduced by 7% compared to R1, which is the net reduction of phasing out fossil fuels and the additional material demand for the sectors’ reconstructions. The remaining fossil fuel carriers in Figure 3a are the use of plastics and the fossil fuel requirements in other sectors, which are not decarbonized in our CeAT model. The increase in biomass use is mainly due to the use of wood for improving windows and roofs during thermal renovation and a small share for wood fuels.

The introduction of weak CE strategies (B1) (Table Table 1) results in a decreased PM by 14% compared to R1. Besides the phasing out of fossil fuels, this is mainly a result of the 25% reduction of per capita floor space in new buildings, which means less material for construction and a lower heating demand. Most other strategies like modal split shifts or improved recycling show minor reductions in material consumption.

Finally, the implementation of a strong set of CE strategies (C1) reduces PM by 72% with respect to R1. This is to a large degree a result of halting the expansion of buildings and road stocks on unbuilt land after 2030 and, to a lesser degree, by the extension of lifetimes for 25% of buildings that are otherwise demolished. Buildings that are finally demolished in this strong CE scenario are replaced at a rate of 50% by buildings mainly made of wood (for impact on biomass use see SI 2.1).

Amongst the three sectors analyzed, the electricity sector has the lowest demand for PM (Figure 3b). Decarbonizing of the electricity sector means phasing out the use of fossil fuels and thus the flow of fuels to power plants. However, the reconstruction of the sector into a green electricity provider still is material intensive in terms of building up required stocks (see SI 2.2). In sum, there is only a slight reduction in PM for the electricity sector between R1 (conventional plants), A1 (decarbonization), and B1 scenario (weak CE). The strong CE scenario (C1) shows reduced material requirements compared to the decarbonization scenario A1 (−17%, Figure 3a). This is due to the lower final energy demand of the transport and building sectors in this scenario, which leads to a lower electricity demand and consequently a lower installed capacity.

### Effects of scenarios on electricity sector and its C emissions

In 2018, the electricity sector generated 266 PJ for final energy use and emitted 0.88 MtC as direct emissions (Figure 3c). Buildings and transport required 546 PJ of final energy consumption, of this, 58 PJ are electricity. In 2040 in the R1 scenario, the electricity sector needs to generate 311 PJ, while in this scenario the buildings and transport sectors still rely largely on fossil fuels.

The significant shift occurs in the A1 scenario, as buildings and transport are decarbonized largely through electrification. Transport needs a strongly increased supply of electricity while buildings have a slightly decreased electricity demand as direct electric heaters are also replaced by heat pumps (Figure 3c). In sum, more electricity is required resulting in rising demand (378 PJ for buildings, transport, and all other sectors). While the demand for electricity increases, the total energy consumption of the building and transport sector is reduced by 32%, especially as there are considerable efficiency gains in the transport sector compared to the R1 scenario, where the combustion of fossil fuels to generate drive energy and electricity is less efficient.

In the A1 scenario, the power supply needs to grow by 40% relative to 2018, but needs to be supplied by green power only in 2040 (Figure 3c). B1 is only easing this task compared to A1 by a minor degree (+33% to 2018). Interestingly, C1 reduces the required electricity generation to the level of the reference scenario. This stems in the first place from the reduced road transport, a combined result of less transport volume for passengers and cargo and a strong modal split shift. While A1, B1, and C1 have no direct C emissions anymore in 2040, the amount of electricity to be generated differs between 308 PJ (C1) and 378 PJ (A1).

Looking at the scenarios in terms of the required carbon budget, only the C1 scenario can remain within the carbon budget attributable to the three sectors (SI 2.3).

### Reduction in material and energy consumption by bundles of strategies

**FIGURE 4:**
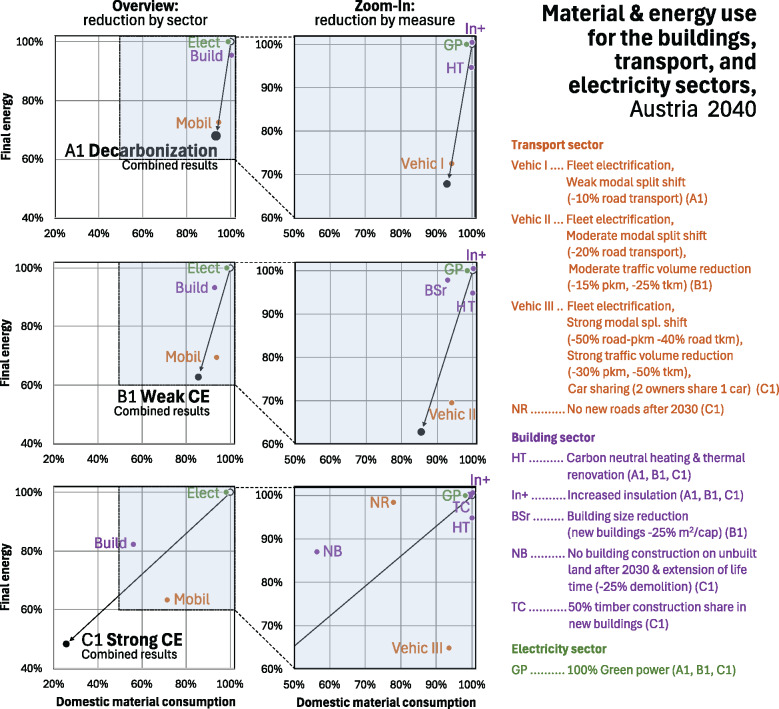
Combined reduction of material and energy use in the buildings, transport, and electricity sector through different bundles of strategies compared to the reference scenario (R1) in 2040 (underlying data are available in SI-data).

If we decompose the material and energy savings of the scenarios for the three sectors by bundle of strategies (Figure 4), it allows us to better understand which bundle can facilitate decarbonization more substantially, and which can contribute more significantly to the CE goals, and which can serve both goals well.

Not surprisingly, A1 reduces energy consumption more strongly (−32%) than DMC (−7%) (Figure 4). The most significant contribution stems from the vehicle electrification and the weak modal split shift away from road transport. A smaller rest can be explained by heating/cooling and insulation strategies, which reduce energy consumption, but DMC reduction due to phasing out fossil fuels is more or less compensated by additional material use for insulation activities.

B1 can reduce both energy (−37%) and material demand (−15%) in a more pronounced way. Again, final energy demand is further reduced mainly by a stronger modal split shift (−31%), but this shift also contributes to reducing DMC (−6%). A slightly stronger DMC reduction stems from the 25% reduction in per capita floor space in new buildings (−7%), but with little energy savings (−2%).

The strong CE scenario C1 is the one with the highest impact on both energy (−52%) and material demand (−74%). In terms of energy the strong modal split shift has the most pronounced effect (−35%), but with moderate material reductions (−6%). No buildings on unbuilt land and reduced demolition allows a substantial reduction of energy (−13%) but more so of material use (−44%). No road construction on unbuilt land has a strong effect on material consumption (−22%), but only minor energy savings (−3%). Even significant reductions in the size of the vehicle fleet do not lead to any noteworthy material reductions, as the maximum stock additions to the fleet in scenario A1 account only for <1% of overall material consumption. Three bundles of strategies together have a very strong combined reduction impact on energy and material: A strong modal split shift, no new roads and no new buildings on unbuilt land explain the overall reduction to a far extent (material: −72% of −74%; energy: −50% of −52%). These high potentials are only to be uncovered through strict narrowing strategies.

### From bulk flows to scarce materials

The scenario impacts on material demand varies for bulk and scarce materials. While bulk material demand decreases from R1 to the decarbonization (A1) and CE scenarios (B1, C1) (Figure 3d), scarce material demand increases significantly from R1 to all other scenarios, as stocks with lower scarce material intensity are replaced by those with higher intensities.

The application of permanent magnets in electric vehicles and in wind power turbines is the main driver for this increased demand. The extensive replacement of conventional with electric vehicles and the increased demand for electricity overall in A1 requires an increase in scarce materials by a factor 9 compared to 2018 and by factor 6 compared to R1 in 2040 (Figure 3d). In B1, the increase compared to R1 in 2040 is only by factor 4 and in C1 only by factor 1.4. The changes are due to fewer vehicles as a result of less road traffic (reduced person and ton kilometers; modal split shift) and car sharing as well as the lower electricity demand with less wind turbines and PV panels.

### Sector results in different economic projections

The different economic projections matter significantly for the DMC as the reference scenario in “slow recovery & zero growth” R2 is well below the “smooth recovery” R1 scenario (−26% points). Only the strong CE (C) matters more than the economic growth effect (−45% points). In other words, without considering other trade-offs (e.g., unemployment and budget deficit), weak economic development can also make the task of reducing material demand to meet the CE strategy targets. This would open up room to allow for adjusting the CE strategies in between the weak and the strong CE scenario while staying on track to the targets (how the scenarios alter Austrian DMC in a time series from 1960 to 2040, see SI 2.4).

### Contribution of narrowing, slowing, and closing loops to scenario results and policy targets

Existing circularity metrics and research commonly lack a comprehensive scope incorporating the hierarchical strategies proposed by the 9R framework (Muñoz et al., 2024). We use the grouping of the CE strategies of the 10R framework in narrowing (highest priority for measures directly reducing resource flows), then slowing (extending lifetimes via reuse, repair, etc.), and finally closing material loops (recycling and recovery) (Morseletto, 2020; N. Zhang et al., 2023; Potting et al., 2017). Here we present the scenario results for the grouped strategies in terms of which of the three strategy approaches contributes to what reduction of the Austrian DMC by 2040 (Figure 6). In the reference scenario (R), recycling, the main closing loop strategy, contributes only moderately with a 10% reduction. In A1, recycling, which is implemented with quite high recycling rates, results in a slightly higher reduction (−11%). Recycling rates in B1 and C1 are again increased as compared to A1, leading to a contribution of recycling at the same level as in A1.

Narrowing flows in A1 leading to a PM reduction of 4% primarily reflect a reduction in energy provisioning, driven by the elimination of fossil fuels and the additional material demand associated with the generation of green electricity. In B1, the effect of narrowing strategies can be doubled to 8% by a stronger shift in transport from road to rail, but mainly by the 25% reduced floor space per capita in new buildings. Finally, a drastic PM reduction is feasible in C1 (−58%). More than half of the saved material is due to stopping construction of new buildings on unbuilt land, and more than a third comes from stopping new road constructions after 2030. Slowing flows can only contribute to a 1% reduction of the overall PM in C1 compared to R1 mainly due to the short period under review and the long service life of stock-building materials dominating mass flows (see SI 2.5). Compared to the PM in R1, recycling can contribute to a 14% reduction of the material demand for primary materials.

If we calculate the socioeconomic input cycling rate (Mayer et al., 2019) or circular material use rate (Eurostat, 2021a), both representing the share of the recycling flow in PM of the respective scenario, they are quite similar for R1, A1, and B1 with 10%, 11%, and 12%, respectively. In C1, the absolute flow of recycling is slightly higher; however, due to the significantly lower amount of PM, the recycling rate is 34%. Thus, narrowing loops have a very positive effect on the socioeconomic input cycling rate.

With regard to the different policy targets, each scenario shows a specific performance (Figure 6). Except for the reference scenario, all scenarios are by design geared toward achieving carbon neutrality by 2040 (BKA, 2020). The goals of the CE strategy (BMK, 2022) are only in reach by the C scenario. Furthermore, the coalition agreement mentions a target of 2.5 ha/day (BKA, 2020), the actual land take is at about 10 ha/day (ÖROK, 2023). In scenario B, a slightly reduced land take can be achieved, as the floor space per capita for new buildings is reduced by 25%, which can even lead to further reductions when combined with redensification. Only in C1, the land take is drastically reduced and in line with the coalition agreements goal as there is no new construction of buildings and roads on unbuilt land.

## DISCUSSION

The results presented here for the achievable material and carbon reductions are well in line with international research on material efficiency and GHG emissions for buildings and vehicles like the review of Hertwich et al. (2019, 2020) or the scenario exercises by Pauliuk et al. (2021) for the world and Pauliuk and Heeren (2020) for Germany. A study on the CE of Australia also highlights similar issues in the construction and transport sectors as they discuss the dematerialization of road construction and the reduction in building sizes as key issues for material reduction (Miatto et al., 2024).

Herein, we discuss for Austria what can be learnt from combining decarbonization with resource use strategies, that is, on changes in stocks and flows in “weak” and “strong CE” scenarios against the background of Austria’s policy targets for decarbonization, CE, and land take.

### Economic development matters more than weak, but less than strong CE initiatives

**FIGURE 5:**
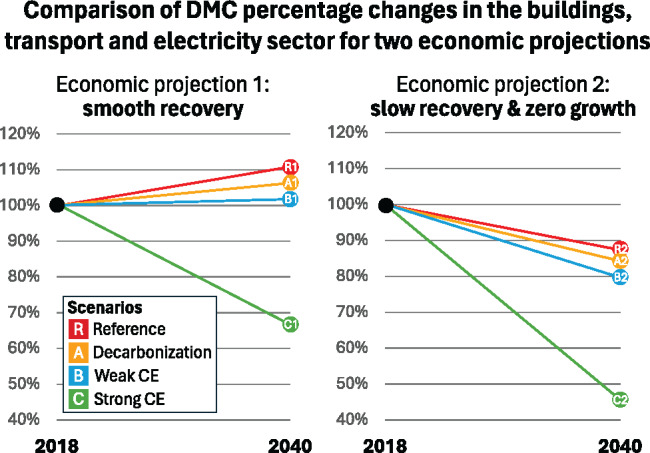
Domestic material consumption (DMC) percentage changes in all three sectors for the scenario reference (R), the decarbonization (A), the “weak circular economy (CE)” (B), and the “strong CE” (C) from 2018 to 2040 for both economic projections (underlying data are available in SI-data).

The results show that a difference of 1.3% in average annual GDP growth has a significant impact on the DMC in 2040. In the scenario with smooth recovery of 1.3% GDP growth, the measures of the strong CE scenario (C1) can reduce DMC by 33%, in the zero growth projection, it is reduced by 54% (C2) however (see Figure 5). Zero growth, thus, makes it easier to achieve the objectives set out in the CE strategy, if other caveats are disregarded. As the Austrian government explicitly strives for economic growth (BKA, 2020), this indicates that economic policies, in particular if they promote growth in the construction sector, undermine CE, and land take targets. Conversely, a policy that takes into account both economic growth and environmental impacts can be successful, for example, if it focuses on incentives for economic activities with low material intensity, in particular the service sector, as was shown in a subsequent analysis (see Meyer et al., 2024).

### Reconstruction for decarbonizing the energy system is not the biggest driver for material demand

The decarbonization in the three sectors is a far-reaching reconstruction program for replacing fossil-fuel-powered stocks like heating systems, cars, and power plants and for refurbishing buildings thermal insulation state. This is a material-intensive undertaking. However, the impact of the omission of fossil fuels to operate processes in the three sectors and its effect on DMC is far higher than what is needed for replacing stocks. For example, the electricity sector saves 1.30 Mt/a fossil fuels and needs 0.13 Mt/a of construction minerals and 0.10 Mt/a metals in an average year in A1 compared to R1 (see Figures 3b and 4). The figures in the fleet electrification are even more pronounced: 5.17 Mt/a of fossil fuels are saved, and 0.13 Mt/a of metals are needed in an average year for reconstruction. On top of this, once reconstruction is finished in 2040, material demand will be reduced to mere maintenance and the replacement of stocks at the end of their service time. The material demand for renewing stocks in the three sectors in an average year is in an order of magnitude of 1 Mt/a. For comparison, the expansion of stocks in the A1 scenario demands 62 Mt/a in 2040. This indicates that the reconstruction of the three sectors is not overstretching the resource demand, and, at the same time, it hints at the potential of policy instruments that can shift construction activities including its financial resources from expansion to reconstruction.

### Recycling’s importance increases with narrowing loops

**FIGURE 6:**
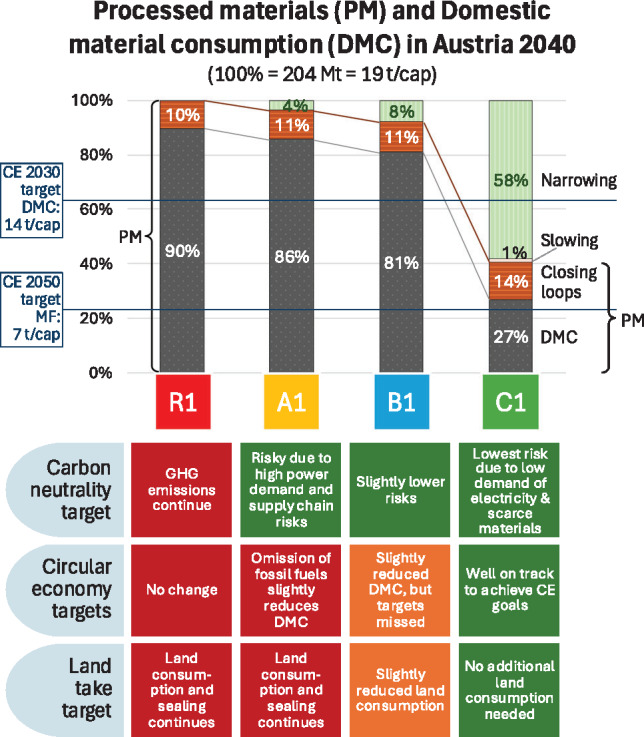
Contributions of narrowing, slowing, and recycling to the reduction of Austrian processed materials (PM) and consequently of domestic material consumption (DMC) in 2040 along four scenarios in the buildings, transport, electricity sector; 100% refers to economy-wide reference scenario PM in 2018; the blue lines represent the 2030 and 2050 CE strategy targets when proportionally applied to the three sectors based on their 2018 DMC and for 2050, assuming a typical Austrian ratio between DMC and material footprint (MF); the boxes below the graph discuss the results of the scenarios in terms of specific policy targets: red boxes indicate that no change toward the target can be detected, orange boxes indicate a change in the desired direction but not strong enough to achieve the target, and green boxes indicate that the targets in these scenarios are within reach (underlying data are available in SI-data).

The CE is often associated with recycling. Here we use a conceptualization that makes use of the full 10R strategies which entail narrowing, slowing, and closing loops, the latter including recycling (Potting et al., 2017). Our results clearly indicate that, in the absence of strong CE measures, the contribution of recycling remains limited (Figure 6). Despite very high end-of-life recycling rates (iron/steel 79% in A and B, 86% in C; construction minerals 92% in A and B, 95% in C) the socioeconomic input recycling rate, that is, the recycling flow in relation to processed material (PM), does not exceed 12%. Only if decisive narrowing strategies substantially reduce PM to 51% compared to the reference scenario, the socioeconomic input cycling rate can be increased to 34%.

### Focusing solely on decarbonization is risky, while a strong CE mitigates implementation risks

A simple decarbonization focus runs the risk that it is to a large extent only feasible through the electrification of the buildings and transport sector and consequently demands a high supply level for growing green power capacities (Elshkaki, 2023; Ghorbani et al., 2024). Although we see that the electrification substantially improves energy efficiency and reduces the overall demand for energy, much more electricity is required in the decarbonization scenario (A) than in 2018 (see Figure 3c). This increase in electricity can only be substantially moderated by narrowing strategies as in the strong CE scenario (C), which are phasing out new construction of buildings and roads on unbuilt land, reduced mileage, and a decisive modal split shift. In particular, the electrification of cars and trucks drives for the enormous rise of electricity demand from less than 3 PJ in the reference scenario R to 68 PJ in the decarbonization scenario A in 2040 (Figure 3c). The strong CE scenario (C) can mitigate electricity demand of the transport sector to 22 PJ in 2040.

It is important to keep electricity demand at check, as the literature shows that permission processes are sensitive and rarely run smooth (Hübner et al., 2023), and as the labor force is not always available at the level of demand to install sufficient PV panels and windmills (Briggs et al., 2022). Finally, the replacement of conventional cars by electric vehicles requires a substantial increase in green power supply. With the additional number of electric vehicles and wind turbines, the demand for permanent magnets increases and thus raises the demand for scarce materials 9-fold compared to 2018 (see Figure 3d). The inclusion of lithium-ion-batteries would further increase the demand for strategic raw materials in decarbonization-focused scenarios and consequently enhance implementation risks, which can be mitigated with strong CE strategies (C) (Helbig et al., 2018). Given the significant risks in supply chains from geopolitical tension or volatile market prices, this makes a transport sector with an exclusive focus on decarbonization very vulnerable and also calls into question whether the government’s climate neutrality goal can be achieved by 2040 at all (C. Zhang et al., 2023; Koide et al., 2022; Papadis & Tsatsaronis, 2020).

A sole focus on decarbonization could limit the scope for action in energy saving. Significant energy savings are possible primarily through low-maintenance and energy-efficient stocks. Thus, the design of the entire transport infrastructure and settlement structures characterized by low sprawl and areas of mixed uses to favor short distance travel in daily activities, are of utmost relevance for mitigating material impacts of the energy transition (Brenner et al., 2024; Cantzler et al., 2020; Creutzig et al., 2012, 2024; Virág et al., 2021).

### Stabilizing the built environment through narrowing and slowing flows is key to meet circular economy and land take goals, while easing decarbonization

The results show that only with far-reaching strategies the goals of carbon neutrality, CE, and land take can be achieved simultaneously (see Figure 6), if the three goals are not pursued in isolation and synergies are realized.

The key to achieving these goals is stabilizing the built environment via the CE strategies narrowing and slowing material flows. First, this reduces energy consumption in extraction and processing of construction materials as well as construction activities itself, which greatly facilitates climate change mitigation; the strong CE scenario (C) uses roughly 20% less of the Austrian carbon budget then the decarbonization (A) and weak CE (B) scenario (Figure SI-8). Second, stabilization of stocks and limited energy consumption means reduced pollution, as this reduces the demand for scarce materials (Figure 3d), which are particularly related to pollution in the mining countries and further along the supply chain (Watari et al., 2020). Third, stabilizing the built environment can halt further land take, which not only poses challenges on its own but also frequently results in fragmentation of landscapes, a key factor contributing for biodiversity loss (Peroni et al., 2022). In conclusion, narrowing strategies can achieve the important outcome of reducing both the scale of the climate challenge and material resource requirements (Creutzig et al., 2024).

### Implications of narrowing and slowing loops for daily life

Whilst some of the CE strategies in C1 may seem incisive, the actual impact on welfare tends to be positive (see Meyer et al., 2024 for an impact analysis of scenarios on value-added, employment, and disposable income) and the quality of life will increase. While car ownership drops dramatically in a strong CE scenario (due to modal shifts and the increase in car sharing), the distance travelled per capita would decrease by 6% compared to 2018. This is justifiable considering that teleworking has recently become increasingly popular, decreasing the need for work-related commuting by car (Hartwig et al., 2022), and since the gradual abandonment of constructing new buildings in the C1 scenario halts further sprawl.

In terms of floor space, we have 72 m^2^/capita for residential, tourism, shop, and office use in 2018, which would increase to 78 m^2^/capita in 2040 in the decarbonization scenario (A1), while it would decrease to 67 m^2^/capita in the strong CE scenario (C1). This reduction in floor space can also be achieved with minor effects for citizens as optimized utilization of existing buildings and halting overtourism have high potentials (SI 3.1).

Thus, depending on the particular implementation, far-reaching changes might not mean sacrifices, but may come with substantial (co-)benefits, which might outweigh the downsides of narrowing flows; however, this is subject to individual preferences. One benefit might be that a shift in the transport sector away from private car ownership increases the disposable income. This calls for accompanying measures to limit rebound effects such as spending on other carbon-intensive activities (see Ornetzeder et al., 2008 for car-free living). An additional benefit is that increased active mobility supports public health by reducing air and noise pollution, while promoting physical activity, which contributes to significant health improvement (Wolkinger et al., 2018). The reduced construction activities (a reduction from 79 [R1] to 14 Mt/a [C1] of non-metallic minerals—Figure 3a) and related reduction in transport of high quantities of material is also reducing traffic, noise, and air pollution.

As the Global Resource Outlook (UNEP IRP, 2024, p. XV) states, these benefits and trade-offs call for “bold policy action to phase out unsustainable activities, speed up responsible and innovative ways of meeting human needs and promote social acceptance of the necessary transitions.”

## CONCLUSION

When discussing the interlinkages between decarbonization and CE, the case of Austria may, by and large, be taken as a typical case for high-income countries, yet, considering the differences in per capita consumption of construction minerals throughout different countries, and the relative high share of renewables in the Austrian electricity sector (SI section 4).

Thus, the Austrian case shows that for the countries of the Global North, the CE may not be a panacea, but it can make decarbonization much more tangible, thereby reducing several implementation risks. Reduced implementation risks are related to the lower demand for green electricity, which is less dependent on smooth approval processes, especially for wind power, and has a lower demand for skilled labor. Further, reduced demand for scarce materials minimizes risks especially when international geopolitical tensions or extreme weather events threaten supply chains.

While a “weak CE” can only make a small contribution in lowering implementation risks of decarbonization, a “strong CE” with a strong reduction in DMC is highly beneficial. However, such a strong CE requires political learning processes on how to move away from long-term patterns of permanent stock expansion and instead reconstruct and stabilize stocks that require significantly less material for maintenance and energy for operation.

It is noteworthy that such a far-reaching transformation is the only scenario for achieving the circularity goals set by the Austrian government. While it involves many changes in daily life, it also has additional benefits, such as health gains from less air and noise pollution and more physical activity in the transport sector.

The scenarios in this research show that especially the demand-side strategies of the CE with a focus on halting expansion of material stocks are key for facilitating the energy transition and keeping material demand at check to lower land take and consequently to effectively address the triple global crisis.

## Supplementary Information

**Supporting Information SI**: This supporting information provides further content as indicated in the main text: on methods (model, scenarios, sectors), results (some more detailed results) and discussion (complementing the text by more supporting information based on literature).

**Supporting Information SI-data**: This supporting information provides overall key data and data as used in the graphs. Supporting information is linked to this article on the *JIE* website.

## Data Availability

The data that support the findings of this study and all data used for the figures are openly available in the provided SI-data.
