# Supplementary material for: How decarbonization and the circular economy interact: Benefits and trade-offs in the case of the buildings, transport, and electricity sectors in Austria
Source: J Ind Ecol. 2025 Jan 31;29(2):531–45. doi: 10.1111/jiec.13619 (PMC13070055; doi:10.1111/jiec.13619)
Supplement: Supplementary file 1 — Supporting Information SI: This supporting information provides further content as indicated in the main text: on methods (model, scenarios, sectors), results (some more detailed results) and discussion (complementing the text by more supporting information based on literature). [file 44498_2025_2902009_MOESM1_ESM.docx]

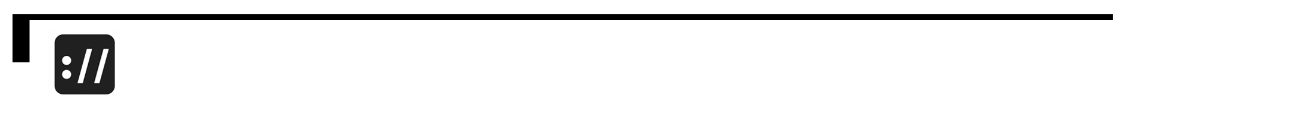


SUPPORTING INFORMATION FOR:

Haas, W., Baumgart, A., Eisenmenger, N., Virág, D., Kalt, G., Sommer, M., Kratena, K., Meyer, I. (2024)

**How decarbonization and the circular economy interact: Benefits and trade-offs in the case of the buildings, transport and electricity sectors in Austria**


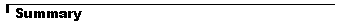


This supporting information provides additional information on the methodology including scenarios, the forecasting method, abbreviations and sectors. Some more detailed results are provided to complement the information in the main text. Also, more detailed background information referring to literature is presented for the discussion section.

Table of Contents

[SUPPORTING INFORMATION FOR: 1](#_Toc182820526)

[1 Methods 3](#_Toc182820527)

[1.1 Reference scenarios (R1, R2) 3](#_Toc182820528)

[1.1.1 Reference scenario R1: Smooth recovery 4](#_Toc182820529)

[1.1.2 Reference scenario R2: Slow recovery and zero growth 4](#_Toc182820530)

[1.2 Forecasting material and energy flows until 2040 5](#_Toc182820531)

[1.3 Sectors 9](#_Toc182820534)

[1.3.1 Buildings 9](#_Toc182820535)

[1.3.2 Transport 10](#_Toc182820536)

[1.3.3 Electricity 12](#_Toc182820537)

[1.4 Decarbonization and CE scenarios 13](#_Toc182820538)

[1.4.1 Scenario overview 13](#_Toc182820539)

[1.4.2 Scenario A: Decarbonization 15](#_Toc182820540)

[1.4.3 Scenario B: Decarbonization and weak CE strategies 15](#_Toc182820541)

[1.4.4 Scenario C: Decarbonization and strong CE strategies 15](#_Toc182820542)

[1.4.5 Scarce materials 16](#_Toc182820543)

[1.5 Additional model parameters 16](#_Toc182820544)

[1.6 Limitations 16](#_Toc182820545)

[2 Results 19](#_Toc182820546)

[2.1 Impact on biomass use due to wood buildings 19](#_Toc182820547)

[2.2 Increased material use for the green electricity sector 19](#_Toc182820548)

[2.3 How the scenarios compare to the carbon budget 19](#_Toc182820549)

[2.4 How scenarios change Austria’s overall DMC in relation to the official domestic circularity targets 20](#_Toc182820550)

[2.5 Impact of disaggregated strategies on final energy use and domestic material consumption 21](#_Toc182820551)

[2.6 Reasons for low potential of slowing loops 22](#_Toc182820552)

[3 Discussion 23](#_Toc182820553)

[3.1 Potentials for reducing heated floor space per capita 23](#_Toc182820554)

[3.2 Options to reduce traffic volume 23](#_Toc182820555)

[4 Conclusions 23](#_Toc182820556)

[5 References 25](#_Toc182820557)


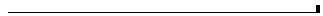


# Methods

The biophysical CE model (CeAT) used in our analysis has previously been developed based on the conceptual framework of economy-wide material flow accounting (MFA). It tracks flows of biomass, metals, non-metallic minerals and fossil materials from extraction and imports into the domestic economy to their processing and conversion into societal material stocks, emissions and waste outputs or recycling inputs. The model has been applied successfully to the global economy, the EU-27, Austria, and South Africa (Haas et al., 2015, 2020; Haas, Virág, Wiedenhofer, & von Blottnitz, 2023; Jacobi et al., 2018; Mayer et al., 2019). For detailed descriptions about the general system and assumptions behind CeAT, we refer to the listed cases. In this study, we based our work on the model for Austria (Jacobi et al., 2018) and extended it in several ways.

First, we updated input data in CeAT to calculate the base year of 2018. We used Eurostat MFA data (Eurostat, 2020a; data download 07/07/2020) on domestic extraction, imports, and exports in Austria in 2018 in kilotons for the 56 material categories reported. We added categories: (a) 4.2.1 ‘Crude oil’ was split into five subcategories (crude oil, plastic, bitumen, lubricants, tires), using information from other data sources and assumptions. (b) 1.7 ‘Cutting from public greens’, which is a flow of biomass not reported in statistics, (c) ‘Extractive Waste’, which is a non-reported flow from metals extraction, (d) ‘Asphalt’, and (e) ‘Concrete’. We introduced relevant compound materials for our case, e.g., concrete or asphalt, and represented the mix of base material to these compounds by introducing a ‘transfer’ of materials (see Haas, Virág, Wiedenhofer, & Von Blottnitz, 2023). Second, we extended the model by a waste module. As the reuse and recycling of materials and the changes in these activities in different scenarios and over time are put in focus in this study, we included a detailed split of waste materials and their fate (reuse, recycling, incineration, disposal, composting). In order to do so, we used waste management data (Eurostat, 2020b) and made informed assumptions to allocate materials from 33 different waste collection categories into the predefined MFA categories. This allowed us to include different categories of waste treatment, i.e. landfills, incineration and energy recovery, recycling and backfilling, and composting, and to allocate waste flows to these.

## Reference scenarios (R1, R2)

To assess the effects of decarbonization and CE scenarios, we developed two reference scenarios (R1, R2), which serve as a reference trajectory up to 2040 from which we can implement at specific points in time the specific scenarios’ bundles of strategies designed in the course of the project. These reference scenarios reflect general developments, such as population growth, changes in GDP, and other variables in line with the principles of the ‘With existing measures’ (WEM) scenario applied by the Federal Environment Agency (Umweltbundesamt, 2023b).

Figure SI-1 Reference scenarios reflecting different economic trajectories following COVID-19

As the project started during the COVID-19 pandemic and the first lockdowns, and further development of the pandemic, the corresponding protective measures, and their economic impacts were highly uncertain, we developed two alternative reference scenarios (R1, R2), reflecting two different growth assumptions until 2040 (see Figure SI-1). The starting point for both scenarios is a pre-pandemic moderate economic growth of 1.5% per year, followed by an economic downturn in 2020 by -6.6% (Schiman-Vukan & Ederer, 2023). After a recovery in 2021/2022 (+4.2%/+4.8%) we assumed an average annual growth rate of 1.33% per year in the reference scenario R1, following the WEM scenario of the Federal Environment Agency (Schiman-Vukan & Ederer, 2023), and, due to interruptions of international value chains, geopolitical tensions and resulting economic frictions, a zero growth rate in the reference scenario R2.

### Reference scenario R1: Smooth recovery

In this scenario we project a steady economic recovery along with the immunization of the population throughout the years 2021ff. The economy was expected to grow by 4.5% in 2021 (Schiman-Vukan & Ederer, 2021) and by 3.5% in 2022. Long-term forecasts for Austria indicate moderate growth rates for the period thereafter. Following the projections by the Federal Environment Agency (Umweltbundesamt, 2021), the annual average growth rates from 2023 to 2030 are about 1.33%. Thus, all economic sectors start their recovery process in the year 2021 and return to the previous growth trajectory with one exception: tourism was projected to lag behind, as global travel warnings were assumed to slow down the recovery process.

### Reference scenario R2: Slow recovery and zero growth

In this scenario we see a long-term structural shift. Due to a stronger impact of the third wave of infections on labor markets and disruptions of international supply chains, the economy is expected to stagnate in the long term with no notable growth in GDP due to ongoing economic frictions. In both scenarios we restrain from explicitly considering increased climate change impacts resulting from an inaction of Austria, considering in principle worldwide climate mitigation.

## Forecasting material and energy flows until 2040

To model the biophysical economy in the reference scenarios, we introduced the time dimension by using the CeAT model to forecast material stocks and flows until 2040. We did this by calculating the historic trend of material intensity (MI) of GDP of the last 15 years (2004-2018) separately for domestic extraction (DE), imports and exports for each of the four main material categories (biomass, fossil fuels, metals, minerals), as displayed in Figure SI-2. We used exponential trends for forecasting, as those delivered the best fit. Based on the value of the last available year (2018) we could thus forecast MI until 2040 for the different indicators (DE, imports, exports) of the four main material groups.

|  | **Domestic Extraction (DE)** | **Imports** | **Exports** |
| --- | --- | --- | --- |
| **Biomass** | 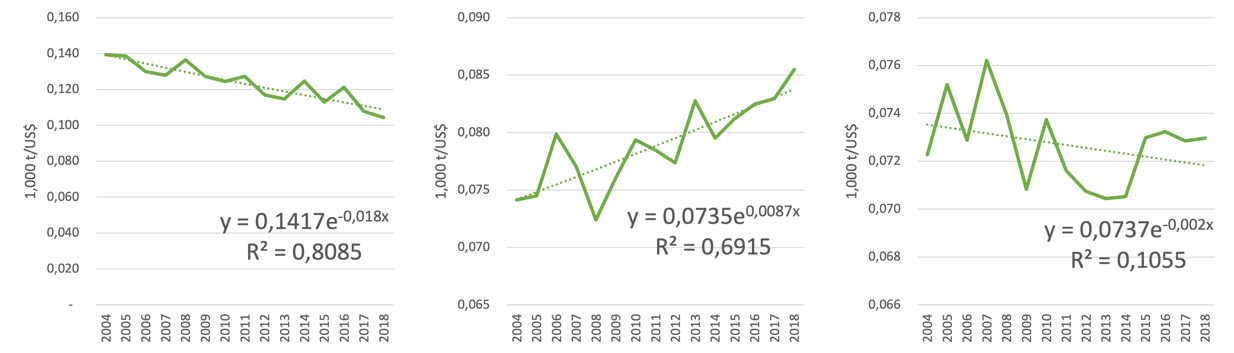 | | |
| **Metals** | 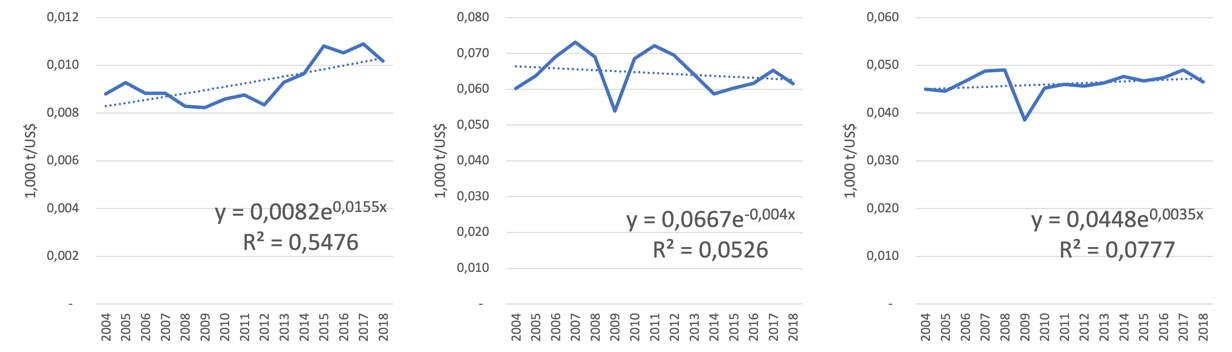 | | |
| **Non-metallic minerals** | 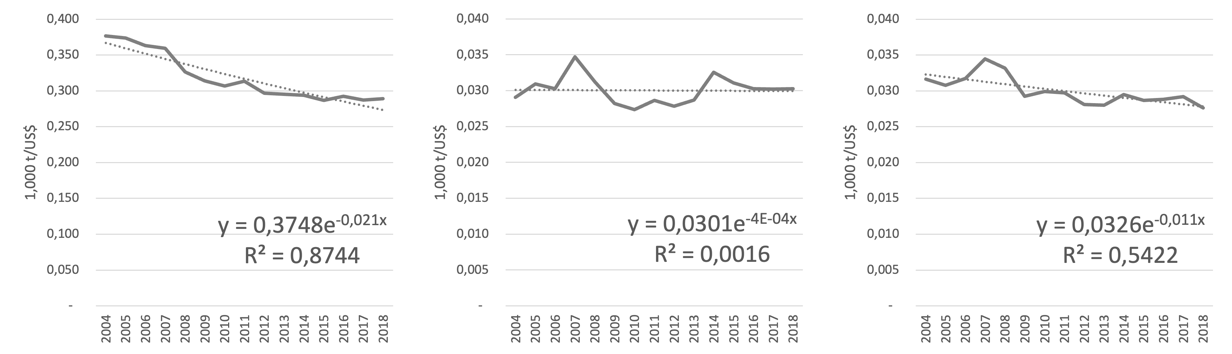 | | |
| **Fossil materials** | 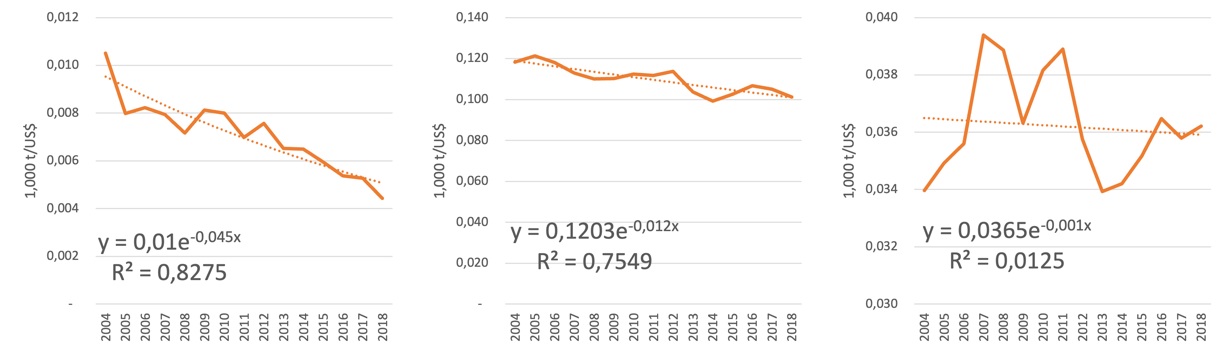 | | |

Figure SI-2 Material intensities of GDP for domestic extraction (DE), imports (Imp) and exports (Exp) for the four main material categories

By multiplying MI with GDP (Eurostat, 2021) for each year, we calculated the material indicators until 2040. For fossil materials we replaced the data with figures forecasted in the WIFO.DYNK model (see Meyer et al., 2024) for total fossil materials and sub-categories, such as coal, oil and gas. The modelled results for all four material categories are displayed in Figure SI-3.


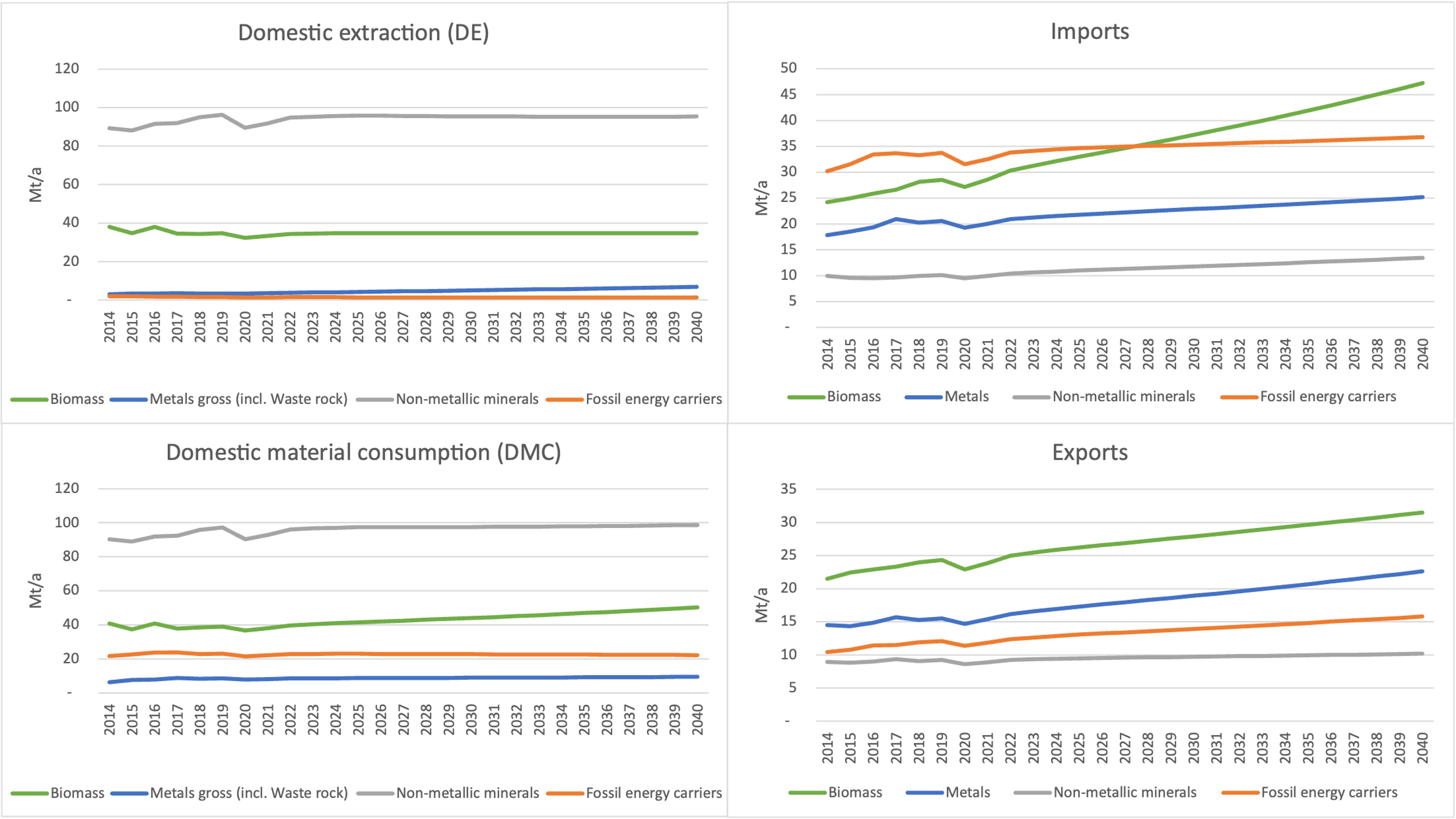


Figure SI-3 Modelled domestic extraction (DE), imports, exports and domestic material consumption (DMC) for the four main material categories until 2040 in reference scenario R1

From the modelled data on DE, imports and exports, we could calculate other flows in our model for every year of the investigation period. We expressed all other flows in the model as percentages of the forecasted values and calculated them for every year, and thus calculated all flows of CeAT for every year until 2040. Table SI-1 lists the main indicators used in the CeAT model and the equations to calculate them.

Table SI-1: List of abbreviations/flows in the CeAT model and equations

| **Abbreviation** | **Flow name** | **Equation or data source** |
| --- | --- | --- |
| DE | Domestic extraction | GDP*MI |
| Imp | Imports | GDP*MI |
| Exp | Exports | GDP*MI |
| PTB | Physical trade balance | imp-exp |
| DMC | Domestic material consumption | DE+imp-exp |
| Transfer | Transfer between categories  (e.g. into compound materials) | % of DMC |
| PM | Processed Materials | DMC+recy+reuse+backfill+transfer |
| MU | Material Use | % of PM |
| EU | Energy Use | % of PM |
| stock add | Additions to stocks | % of MU |
| through | Throughput | MU-stock add - GHG MU |
| GHG from MU | GHG emissions from material use | % of MU |
| dem | Demolition | % of stock add |
| EOL | End of life waste | PM - GHG EU - GHG MU - other em - Vap EU - Vap MU -stockadd +dem- DU II |
| Backfill | Backfilling/downcycling | % of EOL |
| Recy | Recycling | % of EOL |
| Reuse | Reuse | % of EOL |
| GHG (EU) | GHG emissions from energy use | % of EU |
| emissions (other) (S&N, C not inv) | Other emissions; of sulfur, nitrogen and carbon emissions which are not part of the inventory | % of EU |
| Vap MU | Water vapour from material use | % of MU |
| Vap EU | Water vapour from energy use | % of EU |
| waste incin | Waste incineration | % of (EOL-recy-backfill-reuse) |
| energy recov | Energy recovery | % of (EOL-recy-backfill-reuse) |
| em from waste treat (incin/en recov) | Emissions from waste treatment (either incineration or energy recovery) | % of (waste incin + ene recov) |
| vap from waste treat (incin/en recov) | Water vapour from waste treatment (either incineration or energy recovery) | % of (waste incin + ene recov) |
| ash waste incin | Ashes from waste incineration | % of (waste incin + ene recov) |
| direct landfill | Directly landfilled waste | % of EOL |
| compost (EU&MU) | Compost from energy and material use | % of EOL |
| SLO | Solid and liquid outputs | EOL-recy-backfill-reuse-ene recov-waste incin+ash waste incin |
| GHG inv (EU + MU + w.incin) | GHG emissions from inventory (from energy use, material use and waste incineration) | GHG MU + GHG EU + GHG incin |
| vapour (EU + MU + w.incin) | Water vapour from energy use, material use and waste incineration | Vap MU + Vap EU + Vap incin |
| DU I | Dissipative use of materials from energy use (I) | % of EU |
| DU II | Dissipative use of materials from material use (II) | % of MU |

Note that all emissions described in the manuscript are based on emission factors from Krausmann et al. (2018) (see Table SI-5). All energy flows including embodied energy in various stock-building products were multiplied with these emission factors to calculate total emissions caused by material and energy flows.

The following equations were used to ensure that material flows are balanced at the level of main material categories:

- PM = DMC + Backfill + Recy + Reuse
- MU = Stock add + Through + C emissions MU + Vap MU
- EU = S&N emissions + C emissions + Vap EU + Faeces & ashes + Food waste
- EOL = Faeces & ashes – DU I + Food waste + Through – DU II + Dem
- DE + Imp – Exp = DMC = S&N emissions + C emissions + Vap MU + Vap EU + DU I + DU II + SLO + Stock add - Dem

## Sectors

As material stocks are a major driver of material consumption, it was necessary to allocate the current material stock data to their function or end use (e.g., buildings, roads, vehicles) in order to perform prospective scenario modelling. Consequently, three sector modules (building, transport, and electricity production) were developed, collectively covering 51% of emissions and 49% of material consumption (DMC) in Austria in 2018.

For all three sector modules we gathered comprehensive data of the relevant societal stocks, their material and energy requirements (e.g., building types, heating systems, vehicles, roads). Based on these empirical grounds, scenarios reflecting various decarbonization and CE strategies could be developed, with each measure altering future material stocks and consequently material and energy demand and related emissions. After completing these biophysical forecasts, an economic assessment of the different scenarios was conducted using the pre-developed macroeconomic WIFO.DYNK model, including calculations of CE driven macroeconomic rebound effects. The following section describes each working stage in more detail.

### Buildings

In the building sector we considered building construction, demolition, renovation, and heating/cooling system replacement activities and the material inflows and outflows related to these (see Fig. SI-4). In total, four building types (single-family houses, multi-family houses, apartment buildings, commercial buildings), eight building age cohorts (before 1919, 1919 to 1944, 1945 to 1970, 1971 to 1980, 1981 to 1990, 1991 to 2000, 2001 to 2011, and after 2011), and ten heating systems (biomethane, solar, heat pumps, direct electricity, pellet, wood chips, wood logs, natural gas, oil/coal, district heating) were defined.

Figure SI-4 Calculation of building module material inflows and outflows

Data on annually constructed buildings was available via Statistik Austria data (Statistik Austria, 2020b), in the form of number of buildings and square meters constructed. The annually constructed area for residential and non-residential buildings was coupled with population and construction-sector GDP forecasts respectively and forecasted from 2019 to 2040 – analogous to the forecasting approach used for material intensities (see section 1.2).

Starting from building stock as reported in the last 2011 national census (‘Registerzählung 2011’: Statistik Austria, 2011), changes in stock until 2040 were calculated by adding annual new construction and subtracting demolition flows (derived from percentage of stock demolished per year) for Vienna (Lederer et al., 2019). Per age cohort, demolition rates were gradually increased until 2040 and main material outflows were derived.

Building stock thermal renovation, or insulation, rates based on interpolated rates from (Kranzl et al., 2018a) were applied to annual building stock. The thermal renovation rate is understood as the annually thermally renovated floor area per annual total floor area including the area already renovated and newly constructed. Thermal renovation MI factors (kg/m^2^) for outer walls, attic floors and basement ceilings from Maydl (2013) were used in combination with archetypical square meters of building elements for Austria (Austrian Energy Agency, 2011). Rates used for forecasting future thermal renovation in a reference scenario are 1.10% in 2025, 1.02% in 2030, and 0.80% in 2040, with interjacent rates being linearly interpolated. It was assumed that thermal renovation rates only add to material stock with the exception of replaced windows for which average European MI factors for both old and replacement triple-glazed windows were applied. Thermal renovation flows thus add to both material inflows and outflows.

Residential and service sector MI factors for buildings after constructed after 2000, as reported in kg/m^3^ from (Lederer et al., 2021) were converted to kg/building using shares of building volume bins for the total Austrian building stock provided by (Haberl et al., 2021). Multiplied with the number of buildings constructed produces the material inflows (additions to stock) due to new construction.

Final heating energy demand is calculated based on the annual renovated and unrenovated building stock area and age-cohort specific before and after renovation energy demand (kWh/m^2^) (Schulter, 2013). Average material consumption factors of heating system operation are listed in Table SI-2.

Table SI-2 Material consumption factors (calorific value) of different heating systems

| **Heating system** | **Material** | **Unit** | **Value** | **Source** |
| --- | --- | --- | --- | --- |
| Biomethane, H2, synthetic | Crop residue, straw | kWh/kg | 3.50 | Jiang et al., 2019 |
| Heat pumps (air) | Refrigerant | kg/kW/a | 0.03 | Greening & Azapagic, 2012 |
| Heat pumps (ground) | Refrigerant | kg/kW/a | 0.02 |  |
| Heat pumps (water) | Refrigerant | kg/kW/a | 0.02 |  |
| Pellets | Pellets | kWh/kg | 4.72 | Biermayr et al., 2021 |
| Wood chips | Wood chips | kWh/kg | 3.33 |  |
| Wood logs | Wood logs | kWh/kg | 3.97 |  |
| Natural gas | Natural gas | kWh/kg | 10.60 | Forest Research, 2024 |
| Oil and coal | Oil and coal | kWh/kg | 11.80 |  |

After applying heating system shares from Kranzl et al. (2018) and accounting for degree days, the derived installed capacity was multiplied with average heating system MI factors to produce annual heating system material stocks. Positive year-to-year changes add to total material inflows while negative changes add to total outflows. Embodied energy of construction materials, as well as construction, demolition, and renovation activities were calculated based on factors reported in Martínez-Rocamora et al. (2016) and various European LCA case studies.

### Transport

In the transport sector we focused on changes in vehicle fleet size and the extent of the transport infrastructure network and calculated the material inflows and outflows in relation to these (see Fig. SI-5). A variety of vehicles, namely passenger cars, motorcycles, buses, trucks (<3.5t, 3.5-12t, >12t), car and truck trailers, railway (locomotive, rail cars, and coach for both passenger and freight transport), subway (rail cars, coach), trams (rail car, coach), bicycles, and e-bikes were differentiated, as were propulsion types (diesel, gasoline, hybrid, electric, hydro, where applicable). Infrastructure stocks and flows comprise various road (motorway, primary, secondary, tertiary, local, and rural roads) and rail-based (railway lines, subway lines, tram lines) infrastructure types, as well as road and rail bridges and tunnels, and charging stations. Infrastructure stocks and flows comprise both road- and rail-based infrastructure, as well as bridges, tunnels, and charging stations.

For estimating fleet sizes of cars, motorcycles, trucks, and trailers, data from (Statistik Austria, 2020a) was used. For railways, data from Schienen-Control reports (Schienen Control, 2015) was used, while for buses, trams, and subways, regional data from various local transportation companies was combined. For bicycles and e-bikes, data from BMVIT (2013) and VCÖ (2020) reports were used. Historic fleet size data was coupled with European Commission (2020) traffic volume data. To model the vehicle fleet size until 2040, the intensity of the last available year (2018) was multiplied with annual traffic volume which in turn was forecasted by coupling passenger-kilometers (pkm) and ton-kilometers (tkm) to future GDP – analogous to the forecasting approach used for material intensities (see section 1.2). Applying a simple leaching model, end-of-life (EoL) vehicles were calculated based on vehicle lifetime assumptions. Net stock changes (fleet expansion or shrinking) were multiplied with average MI factors from literature, thereby producing material inflows and outflows of the vehicle fleet.

Figure SI-5 Calculation of transport module material inflows and outflows

Austrian infrastructure expansion and maintenance flows were derived from a global transport infrastructure mapping study (Virág, Wiedenhofer, Baumgart, et al., 2022; Wiedenhofer et al., 2024) that spatially maps current and future infrastructure from roads, railway to bridges and tunnels. Charging stations for electric vehicles were modelled using historic ratios of stations to electric vehicles. It is assumed that conventional fossil station infrastructure and hardware can, to a large degree, be used for electric vehicle charging station installations.

The fuel use of vehicles was modeled by converting traffic volume from pkm/tkm to vehicle kilometers (vkm) using occupancy rates (passenger/vehicle/trip) and fuel-use rates listed in Table SI-3. Furthermore, the embodied energy of all materials used in the manufacturing or construction of vehicles and infrastructure was modeled based on factors from literature (Virág, Wiedenhofer, Haas, et al., 2022).

Table SI-3 Energy use per vkm per vehicle type. For the conversion of l/vkm to MJ/vkm the following values were used: gasoline, 32.2 MJ/l; and diesel, 35.9 MJ/l (BMDV, 2014)

| **Vehicle type** | **Fuel type** | **Unit** | **Value** | **Source** |
| --- | --- | --- | --- | --- |
| Cars | Diesel | MJ/vkm | 2.51 | Umweltbundesamt, 2023a |
|  | Gasoline | MJ/vkm | 2.51 | Umweltbundesamt, 2023a |
|  | Hybrid | MJ/vkm | 0.76 | Assumed to be the same as electric cars |
|  | Electric | MJ/vkm | 0.76 | Umweltbundesamt, 2023a |
| Motorcycles | Diesel | MJ/vkm | 1.26 | Assuming a fuel consumption of 3.5 l/vkm |
|  | Electric | MJ/vkm | 0.33 | Weiss et al., 2020 |
| Trucks <3.5 t | Diesel | MJ/vkm | 3.23 | Umweltbundesamt, 2023a |
| Trucks 3.5-12 t | Diesel | MJ/vkm | 6.96 | Umweltbundesamt, 2023a |
| Trucks >12 t | Diesel | MJ/vkm | 11.20 | Umweltbundesamt, 2023a |
| Bus | Diesel | MJ/vkm | 11.24 | Umweltbundesamt, 2023a |
|  | Electric | MJ/vkm | 6.26 | Virág, 2019 |
| Railway | Electric | MJ/vkm | 0.07 | Virág, 2019 |
| Tram | Electric | MJ/vkm | 0.29 | Virág, 2019 |
| Subway | Electric | MJ/vkm | 0.07 | Virág, 2019 |
| E-bike | Electric | MJ/vkm | 0.03 | Weiss et al., 2020 |

### Electricity

Material flows of the electricity production sector are comprised of changes in installed capacity and fuel use (see Fig. SI-6). Electricity use changes from the building and transport modules, and energy sector consumption and transport losses based on static 15-year averages were taken into account when calculating final energy consumption. By applying the last available year’s energy split as static shares, electricity generation per energy system was calculated. By accounting for full-load hours, final energy consumption was converted to installed capacity (MW).

Figure SI-6 Calculation of electricity production module material inflows and outflows

To model future system grid expansion, energy consumption, and installed capacity were integrated with a regression model based on population-coupled household forecasts from Statistics Austria (Statistik Austria, 2020c). The thereby produced <110 kV and >=110 kV system length was then multiplied with the grid type shares (i.e., specific kV bis for overhead and cable) which were forecasted by continuing historic trends to calculate electricity grid system length (circuit km). In addition, electricity grid route length was calculated by dividing system length by the ratio of system to route length for which historic trends were likewise continued.

In a next step, net capacity increases, as well as regular and early decommissioning for both installed capacity in MW and electricity grid system and route length in km were calculated. Regular decommissioning was calculated based on energy system-specific lifetime assumptions ranging from 25 to 50 years, while early decommissioning refers to negative net capacity changes exceeding regular decommissioning. Regular decommissioning was interpreted as decommissioning with replacement, that is the replacement of the power plant or grid section that reached its end of life. Therefore, total installation, are made up of power plant and grid net capacity increases and regular decommissioning. Together, regular and early decommissioning make up total decommissioning. By applying capacity-specific MI factors for steel, concrete, aluminum, and copper (Kalt et al., 2021, 2022), total installation and total decommissioning were converted to material inflows and outflows respectively. Material intensities (t/PJ) were derived from historic data and applied to future electricity generation to calculate fuel use in metric tons for coal, oil, gas, waste, and biomass.

## Decarbonization and CE scenarios

The reference scenario, calculated as described in section 1.1.2, is modified based on multiple assumptions to produce three prospective scenarios ranging from a mere decarbonization of the building, transport, and electricity sectors to combined decarbonization and CE scenarios. With each scenario, the intensity of previously introduced measures is increased and/or new measures are introduced. The relationship between economic trajectories (smooth recovery vs. slow recovery and zero growth) and decarbonization and CE scenario (reference scenario, decarbonization, decarbonization with ‘weak CE’ measures, and decarbonization with ‘strong CE’ measures) are visualized in Figure SI-2.


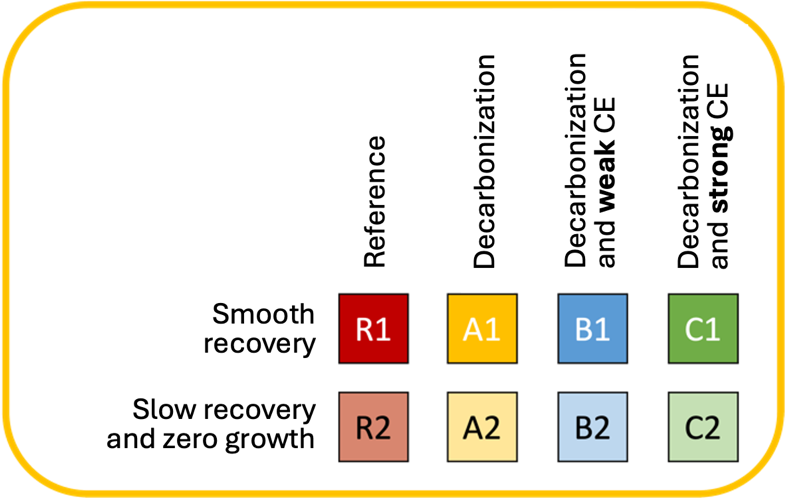


Figure SI-7 Relationship between economic trajectories and decarbonization and CE scenarios

### Scenario overview

Table SI-4 provides an overview of main assumptions used for the implementation of strategies in the three economic sectors for the three prospective scenarios. A more detailed description of scenario assumptions can be found in the subsequent sections 1.4.2-1.4.4.

Table SI-4: Overview of assumptions used in the decarbonization (A), decarbonization and ‘weak CE’ measures (B) and decarbonization and ‘strong CE’ measures (C) scenarios.

| **Sector** | **Measure** | **Assumption** | **Scenario** | **Source** |
| --- | --- | --- | --- | --- |
| Buildings | Decarbonization of heating/cooling systems | 100% phase-out of fossil fuels in heating/cooling by 2040 | A, B, C | Kranzl et al., 2018, österreich.gv.at, 2024 |
|  | Improved thermal renovation rate | Starting with 1.3% of the building stock being thermally renovated in 2018; rate increases to 2.1% in 2030 | A, B, C | Kranzl et al., 2018 |
|  | Increased insulation in new buildings | ~75% increase in insulation material thickness in new construction | A, B, C | Based on Österreichisches Institut für Bautechnik, 2019 |
|  | Reduction of floor space in new buildings | 25% reduction in new per capita floor area | B | Kranzl et al., 2018 |
|  |  | No construction on unbuilt land after 2030 limits floor space in new buildings | C | Own assumption |
|  | Lifetime extension of buildings | Demolition reduced by 25% | C | Own assumption |
|  | Increased timber construction share | Share of timber construction in total construction steadily increased to 50% by 2040 | C | Own assumption |
| Transport | Fleet electrification | 100% electrification of vehicle fleet by 2040 | A, B, C | BMK, 2022 |
|  | Traffic volume reduction by 2040 | -15% pkm,  -25% tkm | B | Own assumption |
|  |  | -30% pkm,  -50% tkm | C | BMVIT, 2014 |
|  | Modal split shift by 2040 | -10% PPV pkm,  -10% tkm road | A | Own assumption |
|  |  | -20% PPV pkm,  -20% tkm road | B | Based on *BMK, 2021:* ‘*Mobilitätsmasterplan’* |
|  |  | -50% PPV pkm,  -40% tkm road | C | Own assumption |
|  | Car fleet size reduction | Reduced in proportion to pkm | B, C | Own assumption |
|  | Car sharing | Only up to 0.01% of cars shared | A, B | BMK, 2023 |
|  |  | Two users share one car in 2040 | C | Own assumption |
|  | Limiting road network development | Expansion halt by 2040 | C | Own assumption |
| Electricity | Fossil fuel phase out | Phase out of coal, oil and natural gas in electricity production | A, B, C | Derived from Austrian Energy Agency, 2017; BMNT, 2019; UBA, 2016, 2023 |
| Economy-wide | Increased recycling rates ^1^ | Iron/steel: 79% | B | Increased recycling for packaging waste share based on Eurostat, 2023 |
|  |  | Plastics: 36% |  |  |
|  |  | Timber: 72% |  |  |
|  |  | Construction minerals: 92% |  |  |
|  |  | Iron/steel: 86% | C | Increase by 30% of recycling gap to 100% (e.g., if rate is 70% in B, it is 80% in C) based on own assumption |
|  |  | Plastics: 57% |  |  |
|  |  | Timber: 81% |  |  |
|  |  | Construction minerals: 95% |  |  |

^1^ Recycling rates for A scenario were not changed and are identical to reference scenario.

### Scenario A: Decarbonization

In the first scenario, future stocks needed for the decarbonization of the three sectors were determined. These are e.g., insulated buildings, heating systems, the vehicle fleet or power plants. For the building sector, heating systems were decarbonized by replacing future reference scenario energy shares with decarbonization scenario energy shares (Kranzl et al., 2018a). The year of complete decarbonization was shifted from 2050 to 2040 (according to the government agreement). Compared to the reference scenario, an increased thermal renovation rate of the existing building stock was assumed, with rates of 1.60% in 2025, 1.98% in 2030 and 1.30% in 2040 (Kranzl et al., 2018a). Reflecting changes in heating energy demand (kWh/m^2^) (OIB, 2019; Österreichisches Institut für Bautechnik, 2007), changes rates for insulation material thicknesses were derived and applied to insulation material MI factors, constituting an increase of 75% on average.

Regarding the transport sector, the vehicle fleet in 2040 was assumed to be fully electrified. For the mobility sector, beyond fleet decarbonization, a slight modal split shift of 10% from cars and motorcycles to rail-based and active mobility and a 10% shift of tkm from road to rail was assumed.

The electricity sector was assumed to be fully decarbonized by 2030, reflecting the ambitious goal of the Austrian government of achieving climate-neutral electricity generation by 2030, ten years prior to EU requirements. Beyond this fossil fuel phase out in electricity generation, no further assumptions were made for the other two prospective scenarios. Additional scenario differences in the electricity sector are instead due to varying electricity demand caused by changes in the other two sectors.

### Scenario B: Decarbonization and weak CE strategies

The second scenario introduces moderate CE strategies. For the building sector, a 25% reduction in newly constructed per capita floor area by 2025 was assumed based on scenario assumptions described in (Kranzl et al., 2018a). This corresponds to a 19% reduction in net floor area in new construction. Reduction was gradually implemented starting in 2018 and kept static after 2025.

In the transport sector, modal split shift was doubled compared to the first scenario with a 20% shift from cars and motorcycles to rail-based and active mobility and a 20% tkm shift from road to rails. An additional traffic volume reduction of 15% for pkm and 25% for tkm was assumed.

### Scenario C: Decarbonization and strong CE strategies

The third and most intense scenario reflects scenarios that go beyond those currently ratified or envisioned by official institutions. For buildings, it was assumed that no construction occurs on previously unbuilt, ‘green’ land. This was implemented by setting the assumption that construction can only occur on land where a building was previously demolished and limiting the annually newly constructed floor area to 50% of the annually demolished floor area. Furthermore, it was assumed that building maintenance and consequently building lifetimes increase, and demolition is reduced by 25%. Finally, it was assumed that 50% of future new construction is using timber substituting for cement/steel, thus changing the MI input and consequently the material composition of the future building stock.

Scenario assumptions for the transport sector include a 50% shift in modal split from cars and motorcycles to rail-based and active mobility, and a 40% tkm shift from road to rail-based transport. Traffic volume reduction was increased to 30% and 50% for pkm and tkm respectively. Furthermore, car sharing was assumed to double by 2040. In regard to transport infrastructure, it was assumed that no further road network expansion occurs after 2030.

### Scarce materials

Beyond bulk-materials, scarce materials (e.g., rare earth elements) widely used in emerging technologies are relevant in the context of resource use and the sustainable transition. To analyze how scarce material demand is impacted by scenarios in contrast to bulk material demand, scarce material demand changes per scenario were calculated in a back-of-the-envelope calculation.

Main scarce materials widely used in scenario-relevant materials were identified based on a literature review: neodymium (Nd), dysprosium (Dy), lanthanum (La), praseodymium (Pr), cerium (Ce), gallium (Ga), germanium (Ge), and tellurium (Te). Using average MI factors from literature (e.g., mg/vehicle component), material stocks of conventional and electronic vehicles, PV, and wind power were estimated. MI factors were compiled in a different research project (Baumgart et al., 2024). Even though so-called technology-critical elements (TCEs) are already used in the manufacturing of conventional vehicles, their application in electric vehicles is significantly higher with an average electric vehicle containing up to 1.3 kg of technology-critical elements in permanent magnets alone (Habib et al., 2020).

Using the same scenario assumptions as for bulk materials (see previous sections), the impact of different measures on the vehicle fleet and PV and wind power capacity, and consequently on scarce materials contained in them, was quantified.

## Additional model parameters

Values in Table SI-5 were used to calculate emissions associated with fuel use. The data was sourced from Krausmann et al. (2018).

Table SI-5 Elements of fuel combustion. All values are % related to kg fuel input fresh weight.

| **Fuel** | **Fuel composition** | | | | | | | **Oxygen  (O_2_ from air)** | **Outputs incl. oxygen from air** | | | | | | | |
| --- | --- | --- | --- | --- | --- | --- | --- | --- | --- | --- | --- | --- | --- | --- | --- | --- |
|  | **Elementary composition** | | | | | **H_2_O moisture** | **Ashes** |  | **Vapor** | | **Solid outputs** | | | **Emissions to air** | | |
|  | **C** | **H2** | **O_2_** | **N** | **S** |  |  |  | **H_2_0 material** | **H_2_0 moisture** | **Ashes** | **S (PM)** | **C** | **CO_2_** | **N_2_O** | **O_2_** |
| Hard coal | 58% | 4% | 7% | 1% | 1% | 21% | 7% | 177% | 38% | 21% | 7% | 1% | 2% | 205% | 2% | 0% |
| Brown coal | 43% | 5% | 13% | 2% | 1% | 32% | 4% | 139% | 46% | 32% | 4% | 1% | 1% | 152% | 3% | 0% |
| Crude oil | 85% | 12% | 0% | 0% | 1% | 0% | 0% | 319% | 106% | 0% | 0% | 1% | 1% | 309% | 1% | 0% |
| Natural gas | 71% | 28% | 0% | 2% | 0% | 0% | 0% | 408% | 246% | 0% | 0% | 0% | 0% | 260% | 2% | 0% |
| Burning wood | 40% | 5% | 35% | 0% | 0% | 15% | 5% | 107% | 43% | 15% | 5% | 0% | 1% | 143% | 0% | 0% |

## Limitations

Our applied methodological approach has certain limitations:

- **Increased climate change impacts not considered:** For all scenarios climate impacts are assumed to be at the same level as in previous years, thus no feedbacks with increasing material demand for repair and restoration are considered.
- **One material intensity trend only:** The projections for the reference scenarios are based on combining material intensities with economic projections. While the economic projections consider the uncertainty of economic development, material intensities follow an exponential trend of the past 15 years without considering different developments. As we mainly use the reference scenarios to see how decarbonization and CE measures alter these reference developments, this assumption has little influence on the robustness of the sector results as all alter the same reference scenarios.
- **Constant proportion of detailed material flows for each main material category in CeAT’s reference scenario:** Projected detailed material flows in the reference scenarios of the CeAT model are considered to follow the same proportional composition of the material main categories as the observed compositions in the recent past. As stocks and related throughput flows were modelled in the three sector modules, there the composition changed. While this is a limitation, it does not alter the robustness of results as CeAT results were only used to check how the aggregate indicator DMC behaves compared to the circular economy targets. Furthermore, for prospective scenarios (A, B, C) this only affects unspecified sectors as stock changes are assumed for the transport, buildings, and electricity sectors and consequently change material flow compositions.
- **Public transport network sufficient for increased use:** As Austria has a very good public transport network which is under constant improvement over the last decade, we assumed that the public transport network is sufficient, and we assumed that we only need to increase the number of trains, trams and busses including the respective energy demand for their operation to deal with the modal split shift which entails increased use of public transport as envisaged in all scenarios albeit to a different degree. We could not finally determine, if this is feasible, but informal expert judgement showed that most likely only little adjustments are needed.
- **Feasibility of increased heating with heat pumps:** Regarding heating, we follow a detailed study on the future of heating in Austria, which has a very good stock taking of buildings, building qualities and heating systems with an energy but no material focus (Kranzl et al., 2018b). The development in this study assumes a high share of heat pumps which we could not scrutinize regarding its feasibility, given that about 60% of the population lives in urban areas in 2023 (UNPD, 2024) where heat pumps are especially challenging. To check this, we would have needed a more spatially explicit modelling which was beyond the scope of our model approach. As the specialized study from sector experts were confident in this assumption, we finally trusted in their judgement.
- **No exact consideration of maintenance flows:** Maintenance is an especially difficult issue as these flows are not easy to be determined for the different stock types. We only considered lifetime extension for buildings. In this case, we would need to model extra flows for refurbishing these buildings with extended lifetimes. We abstained from this, as our maintenance flows are taken from the CeAT model based on the observed past maintenance flows and they are continued to the future. In the scenario C strong CE, the only in which a lifetime extension is envisaged, this means too high a level of maintenance flows, which could, however, cover the renovation required for the lifetime extension. In addition, as we renovate all buildings for thermal insulation, this might include the refurbishing for the lifetime extension as well. However, the main argument, why we did not delve more into this issue, is, that it is a very small number of buildings with extended lifetimes compared to the existing stocks with high levels of maintenance flows. In scenario C strong CE demolished buildings are 0.3% of the entire building stock in 2040. Further, the buildings with lifetime extension are in metric tons only 0.1% of the building stock in this year. Thus, refurbishing flows for the 0.1% of buildings with lifetime extension are negligible and well covered with the overestimated maintenance flows as they are for a growing building stock, however, in scenario C building stocks are kept constant.
- **Only rough comparison of DMC and material footprint (MF) for 2050:** In CeAT we modelled the DMC in t/cap up to 2040, but the circular economy strategy’s target is provided as t/cap MF. To compare DMC and MF for 2050 we made a simple linear extrapolation of the DMC beyond 2040. Further, to compare DMC with MF for 2050 we simply used the same multiplier as in observed data of previous periods between MF/DMC (BMK & BMF, 2024).
- **Land take for PV panels and windmills:** We did not consider the area necessary for additional PV panels and wind turbines. However, PVs need to be as far as possible attached to buildings and infrastructure due to material efficiency reasons. Wind turbines use per MWh more land than fossil-fuel based powerplants, however, compared to other drivers for land consumption they use relatively little land area. The Austrian goal for land take is 2.5 ha/day, the actual consumption is about 10 ha/day (ÖROK, 2023). Land consumption for wind mills are nearly negligible.

In sum, we assessed all these limitations and concluded that they do not jeopardize the robustness of the results as they either apply to all scenarios and thus do not alter them disproportionally with little effect for conclusions derived from a comparison or the respective flows are comparatively small and thus have little consequences for the key results.

# Results

## Impact on biomass use due to wood buildings

Biomass use is in the C1 scenario about the same as it was in 2018, slightly less than in A1, the decarbonization scenario, and the same as in the weak CE scenario B1. The low number of new buildings in the C1 scenario can keep the wood demand at bay. This is even the case with the increased timber construction in C1 (50% of new budlings) due to the low wood demand per conventional building leading to an overall higher wood requirement in the A1 scenario due to the high number of new buildings.

## Increased material use for the green electricity sector

The reconstruction of the electricity sector requires more material than in previous years because of three reasons: First, there is a higher demand per MWh for the construction of green power plants. While gas and oil powerplants require in average about 100 tons of material per MW, while wind turbines and ground-mounted photovoltaic (PV) panels require 650 t/MW and 500 t/MW, respectively. Only roof-top PVs need the low amount of 60 t/MW (Kalt et al., 2021). Second, the installed capacities per produced energy unit need to be higher due to the volatility in production to buffer a “dunkelflaute”, windless days with low sunshine intensity. Third, electricity demand increases in all scenarios except the reference scenario due to the electrification of services in transport and buildings.

## How the scenarios compare to the carbon budget

For Austria a carbon budget of 1,000 MtCO_2e_ was proposed, based on an equal per capita approach applied to the global budget (Kirchengast et al., 2019). Here we simply break down the overall carbon budget of Austria to the three sectors for the period of 2018-2040. To this end we deducted the 2018 CO_2e_-emissions and attributed the budget to the three sectors according to their 2018 share in CO_2e_-emissions. When we use a typical relation for C in CO_2e_ we derive at a carbon budget of about 120 MtC (Fig. SI-8). As the government has agreed to achieve carbon neutrality by 2040, this budget is already applied for the period up to 2040.

Fig. SI-8: Cumulative carbon emissions are shown together with the sectoral carbon budget for the buildings, transport and electricity sector which is estimated to be roughly 120 MtC applying a proportional attribution approach to the sectors buildings, transport, and electricity based on their 2018 share in carbon (C) emissions (see (Kirchengast et al., 2019).

R1 exceeds the carbon budget by 80%, while A1 and B1 are just over the carbon budget (9% respectively 6%). B1 performs slightly better than A1, mainly due to the reduction in new heated floor space and moderate modal split shift, both of which reduce electricity demand and thus carbon emissions during the phase-out of fossil fuel-based electricity generation. C1 is below the budget cap (-10%), with the same mechanisms as B1 coming into play, albeit to a greater extent, as the reduction of heated floor space and modal split shift are more pronounced. While all scenarios achieve carbon neutrality by 2040, A1 and B1 slightly exceed the carbon budget and C1 falls under the budget.

## How scenarios change Austria’s overall DMC in relation to the official domestic circularity targets

The long-term development of the per capita DMC in Austria is characterized by a strong growth phase after 1960 till the early 80s (BMLFUW and BMWFW, 2015). This is followed by stabilization at a level of around 20 t DMC/cap with a few ups and downs. In 2007 the highest per capita value has been achieved with 21 tons/cap. With the financial crisis in 2009/2010 and a slightly stronger population growth the DMC/cap is reduced to about 18 t/cap in 2017/18 (see Fig. SI-9).

The modelled changes in the different scenarios for the three sectors are applied to the overall Austrian material flows. The reference scenario sees still a very slowly growing DMC in the smooth economic projection (R1). The decarbonization (A1) and weak CE (B1) has only a very limited reduction, whereas the strong CE (C1) makes a big impact on the overall DMC development. In the zero growth scenarios (index 2) reduction effects are much stronger.

Fig. SI-9: Development of the Austrian domestic material consumption (DMC) in t/cap/a from 1960 until 2040 in all three decarbonization and CE scenarios (A,B,C) for both reference trajectories: smooth recovery (solid line) and slow recovery, zero growth (dashed line) (Source: BMLFUW and BMWFW, 2015, after 2018: own modelling)

When we focus on the 2030 goal of the CE strategy, A1 and B1 contribute very little to achieve it. A2 and B2 might provide a sufficient reduction, as other sectors need to develop their own reduction strategies. The strong CE scenario in both projections (C1 and C2) can assist the Austrian economy to go below the 14 t/cap DMC goal for 2030 just by the far-reaching CE strategies in the buildings, transport and electricity sector. However, the very ambitious goal of 7 t material footprint per capita can only be achieved with the strong CE scenario if other sectors, not modelled here, contribute their share as well.

## Impact of disaggregated strategies on final energy use and domestic material consumption

An alternative version of Figure 4 in the main document can be found below (Figure SI-4). Here, the impact of each strategy is shown as opposed to the partly aggregated data shown in Figure 4. In particular, strategies related to the vehicle fleet are disaggregated here. These are grouped as ‘Vehic I’, ‘Vehic II’ and ‘Vehic III’ in the main document.

Figure SI-4 Combined reduction of material and energy use in the buildings, transport and electricity sector through different bundles of strategies compared to the reference scenario (R1) in 2040; transport sector strategies disaggregated.

## Reasons for low potential of slowing loops

Slowing loops can only contribute a reduction of -1% of the overall processed materials in C1 compared to R1 mainly due to the short period under review. This has two interwoven reasons: First, the period from 2018 to 2040 is short compared to the long lifetimes of bulky stocks such as buildings and roads, with longer lifetimes only playing out after the period under review. Second, the rebuilding of the sectors makes it necessary to replace fossil-dependent stocks if needed even before the end of their lifetime by those that are based on renewable energies, like power plants, cars and trucks or heating systems. Thus, only the extension of the lifetime of buildings is an option which results in reduced flows. The lifetime extension by +25% of those buildings that would routinely be demolished can yield this -1% reduction.

# Discussion

## Potentials for reducing heated floor space per capita

The older population (60 years and older) is open to a reduction in floor space. For example, a German study found that 29% of homeowners and 11% of tenants are overburdened and consider their living conditions to be too large (Kitzmann, 2023).

The Global Resource Outlook assumes a low utilization rate for office buildings (UNEP, 2024). In a project on existing and planned buildings in Austria, Germany and Switzerland, it was found that buildings are vacant for 90 to 95% of their existence, and strategies and simulation tools were developed to intensify their utilization (Wiegand, 2012). In a Swedish study, meeting room utilization was found to be 14-36% in one specific case, with meeting rooms usually larger than needed and employee attendance in offices below 50% during working times (Holmin et al., 2015).

Another in recent year emerging issue is overtourism. Tourism competes with living space for residents with many adverse effects like unaffordable housing costs for locals or gentrification (see the example of Seville (see the example of Seville Jover & Díaz-Parra, 2023; or detecting overtourism Kirilenko et al., 2023).

A 7% reduction in heated floor space for residents, offices, accommodation and shops on average therefore does not seem to be unrealistic.

## Options to reduce traffic volume

Traffic volume reductions as assumed in the scenarios of this study can be facilitated by various aspects. For example, shifting modes of transportation from private passenger vehicles to active mobility for short distances could greatly reduce motorized traffic volume and consequently the need for vehicle fleet and infrastructure expansion. A study for Austria (BMVIT, 2014) has shown that 17-20% of all trips are at a distance of 2.5 km or lower, while an additional 42-43% of trips are at a distance of 2.5-10 km. While the former distance could be covered on foot, the latter could be covered by bicycle. This highlights that a shift towards resource-efficient (both in terms of fuel use and infrastructure requirements) active mobility can be achieved relatively easy through behavioural changes that would at the same time benefit health and well-being.

More recently, teleworking, or ‘home office’ has become popular. Becoming widespread during the COVID-19 pandemic, working-from-home arrangements allow for reduced commuting requirements. A study for Switzerland has shown that teleworking arrangements during pandemic-related lockdowns have led to a significant decrease of average daily distance travelled for motorized and public transport with increases in active mobility such as cycling (Molloy et al., 2021).

Besides promoting behavioural changes as outlined above, policy changes could greatly benefit reductions in non-active types of mobility as well. Currently, Austria subsidizes diesel fuels with lower taxes for diesel compared to gasoline. This is known as ‘Mineralölsteuer’, colloquially called ‘Dieselprivileg’ (Gass et al., 2014). Likewise, with the Pendlerpauschale, the Austrian government subsidizes work-related commuting (Bundesministerium für Finanzen, 2024). Furthermore, using a milage allowance, company car use is subsidized to a distance travelled of 30.000 km (VCÖ, 2022). Lowing these subsidies and/or instead subsidizing public transport or active mobility could potentially reduce motorized traffic greatly and consequently reduce resource consumption.

# Conclusions

Here we discuss what needs to be considered when using insights from the case of Austria for other economies. Austria is average in terms of its circularity, but has developed a far-reaching circular economy strategy with specific targets. However, China, Japan and the European Union put also a strong emphasis on implementing circular economy approaches to mitigate environmental pressures (Halog & Anieke, 2021).

In this context we can state that Austria is a more or less typical example for a high-income country to investigate how decarbonization and the CE interplay. Within the EU27 Austria has a DMC that is slightly above the region’s average. What needs to be considered is that the metabolic profile shows two special features, namely a relatively high per capita consumption of construction minerals, which is 30% lower in Germany and 40% lower in France and Switzerland in 2022, but it is also 50% higher in Denmark (Eurostat, 2024). The second special feature is a relatively high share of hydro power in the electricity mix which is already for decades around 40% (UBA, 2016b).

Nevertheless, insights from this study are valid for high-income countries in general, especially when considering that interventions into the built environment provide less pronounced impacts in countries with a lower share in per capita use of construction minerals and vice-versa. The reduction potential in the electricity sector for greenhouse gas (GHG) emissions and material consumption is far higher in countries where the green power share is lower, as the phase out of fossil energy carriers has a large effect in both respects.

# References

Austrian Energy Agency. (2011). *TABULA – Eine Typologie österreichischer Wohngebäude*.

Austrian Energy Agency. (2017). *Szenarien für Strom- und Fernwärmeaufbringung im Hinblick auf Klimaziele 2030 und 2050 Endbericht zu Strom und Fernwärme in den Szenarien „WEM“ und „WAM plus“*.

Baumgart, A., Haluza, D., Prohaska, T., Trimmel, S., Pitha, U., Irrgeher, J., & Wiedenhofer, D. (2024). In-use dissipation of technology-critical elements from vehicles and renewable energy technologies in Vienna, Austria: A public health matter? *Journal of Industrial Ecology*. https://doi.org/10.1111/jiec.13571

Biermayr, P., Dißauer, C., Eberl, M., Enigl, M., Fechner, H., Fürnsinn, B., Jaksch-Fliegenschnee, M., Leonhartsberger, K., Moidl, S., Prem, E., Schmidl, C., Strasser, C., Weiss, W., Wittmann, M., Wonisch, P., & Wopienka, E. (2021). *Innovative Energietechnologien in Österreich: Marktentwicklung 2020* (p. 273). https://nachhaltigwirtschaften.at/resources/iea_pdf/marktentwicklung-2020_web.pdf

BMDV. (2014). *Berechnung des Energieverbrauchs und der Treibhausgasemissionen des ÖPNV. Leitfaden zur Anwendung der europäischen Norm EN 16258*. https://bmdv.bund.de/SharedDocs/DE/Anlage/G/energieverbrauch-treibhausgasemission-oepnv.html

BMK. (2021). *Mobilitätsmasterplan 2030 für Österreich*.

BMK. (2022). *Österreich auf dem Weg zu einer nachhaltigen und zirkulären Gesellschaft.* Bundesministerium für Klimaschutz, Umwelt, Energie, Mobilität, Innovation und Technologie. https://www.bmk.gv.at/themen/klima_umwelt/abfall/Kreislaufwirtschaft/strategie.html

BMK. (2023). *Sharing Strategie im Personen-Mobilitätsbereich. Eine Umsetzungsstrategie des Mobilitätsmasterplans 2030 für das Teilen von Fahrzeugen (Sharing) und Fahrten (Mitfahren)*. Bundesministerium für Klimaschutz, Umwelt, Energie, Mobilität, Innovation und Technologie.

BMK, B. für K., & BMF, B. für F. (2024). *Ressourcennutzung in Österreich 2024* (4).

BMLFUW and BMWFW. (2015). *Ressourcennutzung in Österreich, Bericht 2015*.

BMNT. (2019). *Integrierter nationaler Energie- und Klimaplan für Österreich—Periode 2021-2030* (p. 272). https://www.bmlrt.gv.at/dam/jcr:29ba927b-d36f-4cd4-8f56-8bec97a48c76/NEKP_final%2018.12.2019.pdf

BMVIT. (2013). *Radverkehr in Zahlen: Daten, Fakten und Stimmungen*. https://www.bmk.gv.at/themen/mobilitaet/fuss_radverkehr/publikationen/riz.html

BMVIT. (2014). *Österreich unterwegs 2013/2014*.

Bundesministerium für Finanzen. (2024). *Allgemeines zum Pendlerpauschale*. https://www.bmf.gv.at/themen/steuern/arbeitnehmerinnenveranlagung/pendlerfoerderung-das-pendlerpauschale/allgemeines-zum-pendlerpauschale.html

European Commission. (2020). *EU Transport in Figures 2020. Statistical Pocketbook* (Mobility and Transport). Publications Office of the European Union. https://data.europa.eu/data/datasets/eu-transport-in-figures-2020?locale=en

Eurostat. (2020a). *Material flow accounts (env_ac_mfa)* [dataset]. https://ec.europa.eu/eurostat/web/main/data/database

Eurostat. (2020b). *Waste generation and treatment (env_wasgt)* [dataset]. https://ec.europa.eu/eurostat/web/main/data/database

Eurostat. (2021). *GDP and main components (output, expenditure and income)* [dataset]. https://ec.europa.eu/eurostat/databrowser/view/nama_10_gdp__custom_9939779/default/table?lang=en

Eurostat. (2024). *Material Flow Accounts* [dataset]. https://ec.europa.eu/eurostat/databrowser/view/env_ac_mfa/default/table?lang=en&category=env.env_mrp

Eurostat. (2023). *Waste Recycling Targets*. https://ec.europa.eu/eurostat/web/waste/targets

Forest Research. (2024). *Typical calorific values of fuels*. https://www.forestresearch.gov.uk/tools-and-resources/fthr/biomass-energy-resources/reference-biomass/facts-figures/typical-calorific-values-of-fuels/

Gass, V., Schmidt, J., & Schmid, E. (2014). Analysis of alternative policy instruments to promote electric vehicles in Austria. *Renewable Energy*, *61*, 96–101. https://doi.org/10.1016/j.renene.2012.08.012

Greening, B., & Azapagic, A. (2012). Domestic heat pumps: Life cycle environmental impacts and potential implications for the UK. *Energy*, *39*(1), 205–217. https://doi.org/10.1016/j.energy.2012.01.028

Haas, W., Krausmann, F., Wiedenhofer, D., & Heinz, M. (2015). How Circular is the Global Economy?: An Assessment of Material Flows, Waste Production, and Recycling in the European Union and the World in 2005. *Journal of Industrial Ecology*, *19*(5), 765–777. https://doi.org/10.1111/jiec.12244

Haas, W., Krausmann, F., Wiedenhofer, D., Lauk, C., & Mayer, A. (2020). Spaceship earth’s odyssey to a circular economy—A century long perspective. *Resources, Conservation and Recycling*, *163*, 105076. https://doi.org/10.1016/j.resconrec.2020.105076

Haas, W., Virág, D., Wiedenhofer, D., & von Blottnitz, H. (2023). *How Circular is an Extractive Economy? South Africa’s Export Orientation Results in Low Circularity and Insufficient Societal Stocks for Service-Provisioning* (SSRN Scholarly Paper 4386062). https://doi.org/10.2139/ssrn.4386062

Haas, W., Virág, D., Wiedenhofer, D., & Von Blottnitz, H. (2023). *How Circular is an Extractive Economy? South Africa’s Export Orientation Results in Low Circularity and Insufficient Societal Stocks for Service-Provisioning* [Preprint]. SSRN. https://doi.org/10.2139/ssrn.4386062

Haberl, H., Wiedenhofer, D., Schug, F., Frantz, D., Virág, D., Plutzar, C., Gruhler, K., Lederer, J., Schiller, G., Fishman, T., Lanau, M., Gattringer, A., Kemper, T., Liu, G., Tanikawa, H., Van Der Linden, S., & Hostert, P. (2021). High-Resolution Maps of Material Stocks in Buildings and Infrastructures in Austria and Germany. *Environmental Science & Technology*, *55*(5), 3368–3379. https://doi.org/10.1021/acs.est.0c05642

Habib, K., Hansdóttir, S. T., & Habib, H. (2020). Critical metals for electromobility: Global demand scenarios for passenger vehicles, 2015–2050. *Resources, Conservation and Recycling*, *154*, 104603. https://doi.org/10.1016/j.resconrec.2019.104603

Halog, A., & Anieke, S. (2021). A Review of Circular Economy Studies in Developed Countries and Its Potential Adoption in Developing Countries. *Circular Economy and Sustainability*, *1*(1), 209–230. https://doi.org/10.1007/s43615-021-00017-0

Holmin, J., Levison, E., & Oehme, S. (2015). *The utilization of office spaces and its impact on energy use*.

Jacobi, N., Haas, W., Wiedenhofer, D., & Mayer, A. (2018). Providing an economy-wide monitoring framework for the circular economy in Austria: Status quo and challenges. *Resources, Conservation and Recycling*, *137*, 156–166. https://doi.org/10.1016/j.resconrec.2018.05.022

Jiang, Y., Havrysh, V., Klymchuk, O., Nitsenko, V., Balezentis, T., & Streimikiene, D. (2019). Utilization of Crop Residue for Power Generation: The Case of Ukraine. *Sustainability*, *11*(24), Article 24. https://doi.org/10.3390/su11247004

Jover, J., & Díaz-Parra, I. (2023). Who is the city for? Overtourism, lifestyle migration and social sustainability. In *Migration, Tourism and Social Sustainability*. Routledge.

Kalt, G., Thunshirn, P., Krausmann, F., & Haberl, H. (2022). Material requirements of global electricity sector pathways to 2050 and associated greenhouse gas emissions. *Journal of Cleaner Production*, *358*, 132014. https://doi.org/10.1016/j.jclepro.2022.132014

Kalt, G., Thunshirn, P., Wiedenhofer, D., Krausmann, F., Haas, W., & Haberl, H. (2021). Material stocks in global electricity infrastructures – An empirical analysis of the power sector’s stock-flow-service nexus. *Resources, Conservation and Recycling*, *173*, 105723. https://doi.org/10.1016/j.resconrec.2021.105723

Kirchengast, G., Kromp-Kolb, H., Steininger, K., Stagl, S., Kirchner, M., Ambach, C., Grohs, J., Gutsohn, A., Peisker, J., & Strunk, B. (2019). *Referenzplan als Grundlage für einen wissenschaftlich fundierten und mit den Pariser Klimazielen in Einklang stehenden Nationalen Energie-und Klimaplan für Österreich (Ref- NEKP)*. Climate Change Centre Austria. https://ccca.ac.at/fileadmin/00_DokumenteHauptmenue/03_Aktivitaeten/UniNEtZ_SDG13/RefNEKP/Ref-NEKP_Gesamtdokument_PublVers-9.9.2019.pdf

Kirilenko, A. P., Ma, S. (David), Stepchenkova, S. O., Su, L., & Waddell, T. F. (2023). Detecting Early Signs of Overtourism: Bringing Together Indicators of Tourism Development With Data Fusion. *Journal of Travel Research*, *62*(2), 382–398. https://doi.org/10.1177/00472875211064635

Kitzmann, R. (2023). Home swapping as instrument for more housing sufficiency! *International Journal of Housing Policy*, 1–18. https://doi.org/10.1080/19491247.2023.2269619

Kranzl, L., Müller, A., Maia, I., Büchele, R., & Hartner, M. (2018a). *Wärmezukunft 2050. Erfordernisse und Konsequenzen der Dekarbonisierung von Raumwärme und Warmwasserbereitstellung in Österreich*. TU Wien.

Kranzl, L., Müller, A., Maia, I., Büchele, R., & Hartner, M. (2018b). *Wärmezukunft 2050. Erfordernisse und Konsequenzen der Dekarbonisierung von Raumwärme und Warmwasserbereitstellung in Österreich. Kurzfassung*. Technische Universität Wien, Energy Economics Group. https://eeg.tuwien.ac.at/fileadmin/user_upload/projects/import-downloads/PR_469_Waermezukunft_2050_Kurzfassung.pdf

Krausmann, F., Lauk, C., Haas, W., & Wiedenhofer, D. (2018). From resource extraction to outflows of wastes and emissions: The socioeconomic metabolism of the global economy, 1900–2015. *Global Environmental Change*, *52*, 131–140. https://doi.org/10.1016/j.gloenvcha.2018.07.003

Lederer, J., Fellner, J., Gassner, A., Gruhler, K., & Schiller, G. (2021). Determining the material intensities of buildings selected by random sampling: A case study from Vienna. *Journal of Industrial Ecology*, *25*(4), 848–863. https://doi.org/10.1111/jiec.13100

Lederer, J., Gassner, A., Keringer, F., Mollay, U., Schremmer, C., & Fellner, J. (2019). Material Flows and Stocks in the Urban Building Sector: A Case Study from Vienna for the Years 1990–2015. *Sustainability*, *12*(1), 300. https://doi.org/10.3390/su12010300

Martínez-Rocamora, A., Solís-Guzmán, J., & Marrero, M. (2016). LCA databases focused on construction materials: A review. *Renewable and Sustainable Energy Reviews*, *58*, 565–573. https://doi.org/10.1016/j.rser.2015.12.243

Maydl, P. (2013). *Möglichkeiten und Grenzen von Gebäudesanierungen auf Plusenergiehausstandard Know-How-Plus*. BMVIT.

Mayer, A., Haas, W., Wiedenhofer, D., Krausmann, F., Nuss, P., & Blengini, G. A. (2019). Measuring Progress towards a Circular Economy: A Monitoring Framework for Economy-wide Material Loop Closing in the EU28. *Journal of Industrial Ecology*, *23*(1), 62–76. https://doi.org/10.1111/jiec.12809

Meyer, I., Sommer, M., Kratena, K., Baumgart, A., Eisenmenger, N., & Haas, Wi. (2024). Dekarbonisierung und Kreislaufwirtschaft Ökonomische und biophysische Effekte verschiedener Szenarien für Österreich. *WIFO Monatsberichte (Monthly Reports)*, 16.

Molloy, J., Schatzmann, T., Schoeman, B., Tchervenkov, C., Hintermann, B., & Axhausen, K. W. (2021). Observed impacts of the Covid-19 first wave on travel behaviour in Switzerland based on a large GPS panel. *Transport Policy*, *104*, 43–51. https://doi.org/10.1016/j.tranpol.2021.01.009

OIB, Ö. I. für B. (2019). *OIB-Richtlinien 2019*. https://www.oib.or.at/oib-richtlinien/richtlinien/2019

ÖROK. (2023). *Bodenstrategie für Österreich—Strategie zur Reduktion der weiteren Flächeninanspruchnahme und Bodenversiegelung bis 2030*.

österreich.gv.at. (2024). *“raus aus Öl und Gas” 2023/2024*. https://www.oesterreich.gv.at/themen/umwelt_und_klima/energie_und_ressourcen_sparen/1/raus_aus_oel.html

Österreichisches Institut für Bautechnik. (2007). *OIB-Richtlinien 2007*. https://www.oib.or.at/de/oib-richtlinien/richtlinien/2007

Schienen Control. (2015). *Jahresbericht 2015*. https://www.schienencontrol.gv.at/de/Archiv.html?file=files/1-Homepage-Schienen-Control/1f-Publikationen/

Schiman-Vukan, S., & Ederer, S. (2021). *Prognose für 2021 und 2022: Vierte COVID-19-Welle bremst kräftigen Aufschwung, WIFO-Konjunkturprognose*. Österreichisches Institut für Wirtschaftsforschung.

Schiman-Vukan, S., & Ederer, S. (2023). *WIFO Konjunkturprognose 3/2023, Kaufkraft steigt nach milder Rezession. Prognose für 2023 und 2024*. Österreichisches Institut für Wirtschaftsforschung.

Schulter, D. (2013). *Nachhaltige Gebäudesanierung durch lebenszyklusorientierte Bauproduktauswahl*.

Statistik Austria. (2011). *Registerzählung 2011*.

Statistik Austria. (2020a). *Kfz-Bestand* [dataset]. https://www.statistik.at/statistiken/tourismus-und-verkehr/fahrzeuge/kfz-bestand

Statistik Austria. (2020b). *Wohnungs- und Gebäudeerrichtung – Fertigstellungen* [dataset]. https://www.statistik.at/web_de/statistiken/menschen_und_gesellschaft/wohnen/wohnungs_und_gebaeudeerrichtung/fertigstellungen/index.html

Statistik Austria. (2020c). *Haushaltsprognosen*. https://www.statistik.at/statistiken/bevoelkerung-und-soziales/bevoelkerung/familien-haushalte-lebensformen/haushaltsprognosen

UBA. (2016a). *Szenario erneuerbare Energie 2030 und 2050.* (REP-0576). Umweltbundesamt. https://www.umweltbundesamt.at/fileadmin/site/publikationen/rep0576.pdf

UBA. (2016b). *Szenario erneuerbare Energie 2030 und 2050*.

UBA. (2023). *Energie- und Treibhausgasszenarien 2023: WEM, WAM und Transition mit Zeitreihen von 2020 bis 2050* (REP-0882). Umweltbundesamt. https://www.umweltbundesamt.at/fileadmin/site/publikationen/rep0882.pdf

Umweltbundesamt. (2021). *GHG Projections and Assessment of Policies and Measures in Austria*.

Umweltbundesamt. (2023a). *Emissionskennzahlen 2022*. https://www.umweltbundesamt.at/fileadmin/site/themen/mobilitaet/daten/ekz_doku_verkehrsmittel.pdf

Umweltbundesamt. (2023b). *GHG Projections and Assessment of Policies and Measures in Austria*.

UNEP. (2024). *Global Resources Outlook 2024: Bend the trend. Pathways to a liveable planet as resource use spikes*. https://www.unep.org/resources/Global-Resource-Outlook-2024

UNPD, U. N. P. D. (2024). *World Urbanization Prospects: 2018 Revision.* [dataset].

VCÖ. (2022). *Kilometergeld und Dienstwagen-Privileg zu Mobilitätsbudget weiterentwickeln*. https://vcoe.at/themen/kilometergeld-und-dienstwagen-privileg-zu-mobilitaetsbudget-weiterentwickeln

VCÖ. (2020). *Bereits mehr als 750.000 E-Fahrräder in Österreich – E-Fahrräder klarer Spitzenreiter unter den E-Fahrzeugen*. https://www.vcoe.at/presse/presseaussendungen/detail/vcoe-bereits-mehr-als-750-000-e-fahrraeder-in-oesterreich-e-fahrraeder-klarer-spitzenreiter-unter-den-e-fahrzeugen

Virág, D. (2019). Das Verkehrssystem im Stock-Flow-Service-Nexus. Analyse der Materialbestände und -flüsse für verschiedene Formen von Mobilität in Wien. *Social Ecology Working Paper*, *179*.

Virág, D., Wiedenhofer, D., Baumgart, A., Matej, S., Krausmann, F., Min, J., Rao, N. D., & Haberl, H. (2022). How much infrastructure is required to support decent mobility for all? An exploratory assessment. *Ecological Economics*, 14. https://doi.org/10.1016/j.ecolecon.2022.107511

Virág, D., Wiedenhofer, D., Haas, W., Haberl, H., Kalt, G., & Krausmann, F. (2022). The stock-flow-service nexus of personal mobility in an urban context: Vienna, Austria. *Environmental Development*, *41*, 100628. https://doi.org/10.1016/j.envdev.2021.100628

Weiss, M., Cloos, K. C., & Helmers, E. (2020). Energy efficiency trade-offs in small to large electric vehicles. *Environmental Sciences Europe*, *32*(1), 46. https://doi.org/10.1186/s12302-020-00307-8

Wiedenhofer, D., Baumgart, A., Matej, S., Virág, D., Kalt, G., Lanau, M., Tingley, D. D., Liu, Z., Guo, J., Tanikawa, H., & Haberl, H. (2024). Mapping and modelling global mobility infrastructure stocks, material flows and their embodied greenhouse gas emissions. *Journal of Cleaner Production*, *434*, 139742. https://doi.org/10.1016/j.jclepro.2023.139742

Wiegand, D. (2012). MoreSpace—Organisation der Raumnutzung über die Zeit. In *Stadt: Gestalten: Festschrift für Klaus Semsroth* (pp. 199–204). Springer. https://doi.org/10.1007/978-3-7091-1057-7_37
